# Understanding the implementation of Direct Health Facility Financing and its effect on health system performance in Tanzania: a non-controlled before and after mixed method study protocol

**DOI:** 10.1186/s12961-018-0400-3

**Published:** 2019-01-30

**Authors:** Ntuli A. Kapologwe, Albino Kalolo, Stephen M. Kibusi, Zainab Chaula, Anna Nswilla, Thomas Teuscher, Kyaw Aung, Josephine Borghi

**Affiliations:** 1 Department of Health, Social welfare and Nutrition Services, President’s Office Regional Administration and Local Government (PORALG), P.O Box 1923, Dodoma, Tanzania; 2 Department of Community Health, St. Francis University College of Health and Allied Sciences, P.O Box 175, Ifakara, Tanzania; 3 College of Health Sciences, School of Nursing and Public Health, University of Dodoma, P.O Box 395, Dodoma, Tanzania; 4 President’s Office Regional Administration and Local Government (PORALG), P.O Box 1923, Dodoma, Tanzania; 5 Department of Health, Social welfare and Nutrition Services, President’s Office Regional Administration and Local Government (PORALG), P.O Box 1923, Dodoma, Tanzania; 6 Embassy of Switzerland, P.O Box 23371, Dar Es Salaam, Tanzania; 7 Unicef –Tanzania, P.O Box 4076, Dar Es Salaam, Tanzania; 8 Department of Global Health and Development, London School of Hygiene & Tropical Medicine, 15-17 Tavistock Place, London, WC1H 9SH United Kingdom

**Keywords:** Direct Health Facility Financing, health system performance, structural quality of healthcare, health system responsiveness, implementation fidelity, primary healthcare facilities, Tanzania

## Abstract

**Background:**

Globally, good health system performance has resulted from continuous reform, including adaptation of Decentralisation by Devolution policies, for example, the Direct Health Facility Financing (DHFF). Generally, the role of decentralisation in the health sector is to improve efficiency, to foster innovations and to improve quality, patient experience and accountability. However, such improvements have not been well realised in most low- and middle-income countries, with the main reason cited being the poor mechanism for disbursement of funds, which remain largely centralised. The introduction of the DHFF programme in Tanzania is expected to help improve the quality of health service delivery and increase service utilisation resulting in improved health system performance. This paper describes the protocol, which aims to evaluate the effects of DHFF on health system performance in Tanzania.

**Methods:**

An evaluation of the effect of the DHFF programme will be carried out as part of a nationwide programme rollout. A before and after non-controlled concurrent mixed methods design study will be employed to examine the effect of the DHFF programme implementation on the structural quality of maternal health, health facility governing committee governance and accountability, and health system responsiveness as perceived by the patients’ experiences. Data will be collected from a nationally representative sample involving 42 health facilities, 422 patient consultations, 54 health workers, and 42 health facility governing committees in seven regions from the seven zones of the Tanzanian mainland. The study is grounded in a conceptual framework centered on the Theory of Change and the Implementation Fidelity Framework. The study will utilise a mixture of quantitative and qualitative data collection tools (questionnaires, focus group discussions, in-depth interviews and documentary review). The study will collect information related to knowledge, acceptability and practice of the programme, fidelity of implementation, structural qualities of maternal and child health services, accountability, governance, and patient perception of health system responsiveness.

**Discussion:**

This evaluation study will generate evidence on both the process and impact of the DHFF programme implementation, and help to inform policy improvement. The study is expected to inform policy on the implementation of DHFF within decentralised health system government machinery, with particular regard to health system strengthening through quality healthcare delivery. Health system responsiveness assessment, accountability and governance of Health Facility Government Committee should bring autonomy to lower levels and improve patient experiences. A major strength of the proposed study is the use of a mixed methods approach to obtain a more in-depth understanding of factors that may influence the implementation of the DHFF programme. This evaluation has the potential to generate robust data for evidence-based policy decisions in a low-income setting.

**Electronic supplementary material:**

The online version of this article (10.1186/s12961-018-0400-3) contains supplementary material, which is available to authorized users.

## Background

There is a growing recognition of the need to understand the implementation of complex public health interventions within programme evaluation. Implementation indicators also need to be linked to measurements of programme impact [1–3]. Assessing the extent to which programme implementation adhered to the design, together with identifying implementation challenges and bottlenecks, can help to determine if a lack of programme effect relates to a programme being ineffective or to a failed implementation process [4]. Moreover, an understanding of the implementation processes can contribute to supporting internal and external validity of the intervention. Specifically, assessing the implementation process of complex health interventions helps to (1) provide feedback for improving the programme, (2) replicate the programme in other settings, (3) interpret the impact of the programme, and (4) appraise the generalisability and transferability of the programme [5].

Many sub-Saharan African countries have been introducing reforms to improve their primary healthcare and referral systems [6, 7]. These reforms are typically complex by nature, and aim to strengthen and transform the health system by targeting specific health system inputs. One such approach is the introduction of fiscal decentralisation through directing health facility financing to primary healthcare providers [8], with the aim of granting autonomy to these providers in the planning, management and use of funds [9]. Such an approach was recently implemented in Kenya, where initial assessment has shown some positive results in terms of increased autonomy by frontline workers and improvements in governance and accountability [10, 11]. Inspired by this experience, Tanzania has just embarked on a Direct Health Facility Financing (DHFF) programme with a view to improving the performance of its primary healthcare system [12–15].

Although some primary healthcare facilities in Tanzania have already been exposed to financial autonomy linked to other health programmes (such as Results-Based Financing and the Community Health Fund (a community-based health insurance scheme)), the current reform is the first national initiative to scale-up financial autonomy for primary healthcare providers.

Evaluations of fiscal decentralisation in the health sector in other countries have lacked emphasis regarding how such reforms have affected health system outcomes. Systematic assessment of the implementation processes of the reforms has also been lacking [16–21]. Moreover, in sub-Saharan African settings, there is limited evidence about process evaluations that combine both impact evaluation and assessment of the implementation processes, in particular considering the fact that DHFF is a new concept on trials within the region [22, 23].

In light of the limited evidence regarding outcome and process evaluation of DHFF programme implementation in primary healthcare facilities, this paper presents a protocol for an evaluation of implementation fidelity of the DHFF programme and its mechanisms of effect. It specifically focuses on its impact on health system performance in Tanzania. This is in line with the Medical Research Council guidelines for process evaluation [24, 25]. More specifically, this study will be examining the effect of the DHFF programme on the structural quality of maternal health, Health Facility Governing Committee Governance (HFGCs) and accountability, and health system responsiveness as perceived by patient experience. In addition to examining factors facilitating DHFF implementation, the study is set out to document unintended consequences and to provide feedback to implementers concerning how to improve programme performance.

## Methods

### Research setting

Since independence (in 1961), coordination and implementation of all health services in Tanzania has been under the Ministry of Health, currently known as the Ministry of Health, Community Development, Gender, Elderly and Children. Following sectoral reforms, currently, this Ministry is mainly responsible for health policy and the formulation of guidelines [12]. The Department of Health, Social Welfare, and Nutrition Services in the President’s Office Regional Administration and Local Government is responsible for the interpretation of policies and coordination of policy implementation at the Regional and Local Government Authorities. There is a decentralised structure of management of health services with the Regional Health Management Teams at the regional level holding responsibility for conducting supportive supervision and mentorship for the district councils on issues related to the DHFF programme implementation. The Council Health Management Teams (CHMT) at district level will be responsible for ensuring that the DHFF programme is implemented according to the design. They will also hold responsibility for providing technical assistance to the primary healthcare facilities about DHFF programme implementation, including financial management and preparation of annual plans and budget for the individual health facilities. The Facility Management Teams and HFGCs will have responsibility for planning and budgeting for the health facilities in addition to endorsement of all transactions at these primary health facilities. The HFGCs were introduced in 1999 and consist of five members of the community and three appointed members, namely the health facility in-charge, a member of the Village Government committee and a member of the Ward Development Committee. The committees meet four times a year. Their main roles and responsibilities are safeguarding the effective use of resources, ensuring the smooth operation of health facility activities, and developing facility plans and budgets. Other roles include mobilising the community to contribute to the community health fund (a community-based health insurance scheme) and ensuring the availability of medicines and equipment within the health facilities [26].

The HFGCs are also responsible for endorsing reports generated from the Facility Financial and Reporting System before being submitted to the district council and the regional secretariat [12].

The CHMT, which includes 16 members [27], is responsible for planning and budgeting, managing human resources for health, mobilisation of resources at the local level, conducting supportive supervision, mentorship, evaluation and coordination of all health-related activities at district level (including primary healthcare facilities). A further accountability structure is the Council Health Service Board, a team composed of members from the District council and the community. They are responsible for mobilising resources necessary for implementing health interventions at the district level [28]. The Council Health Service Boards act as the think-tank for creativity and innovations for health at the district level. The board includes elected councillors, who may also chair the social service (Health, Water and Education sectors) committees. These boards are responsible for setting up the vision for health services and programmes in the district and work to mobilise resources for smooth implementation of various activities and interventions. In this regard, the boards are responsible for analysing problems, setting priorities, and approving budgets and plans at the district level.

The Tanzanian health system is funded mainly through government sources (revenue collected from income tax and value added tax, donor contributions), grants and loans for health programmes, pre-payment schemes (i.e. social health insurance schemes, the community health fund and private health insurance) and out-of-pocket contributions (direct payments when accessing services). The main pre-payment schemes are the National Health Insurance Fund, the National Social Security Fund and the Community Health Fund [29, 30].

The Health Basket Fund (a grant by donors) has been financing the health sector in Tanzania since 1999/2000. It is part of the government effort to implement a sector-wide approach arrangement where different development partners put their financial contributions into one basket and then support the health sector through 13 priority areas as spelled out in the Comprehensive Council Health Plan and Comprehensive Health Plans guidelines [31]. It is considered one of the most reliable sources of funds in the country. Currently, there are six development partners contributing to the fund. The release of these funds is guided by signing of the contract after mutual agreement between Health Basket Financing partners and the Government of Tanzania. It is a part of the implementation of the 5-year Memorandum of Understanding [32].

**Fig. 1:**
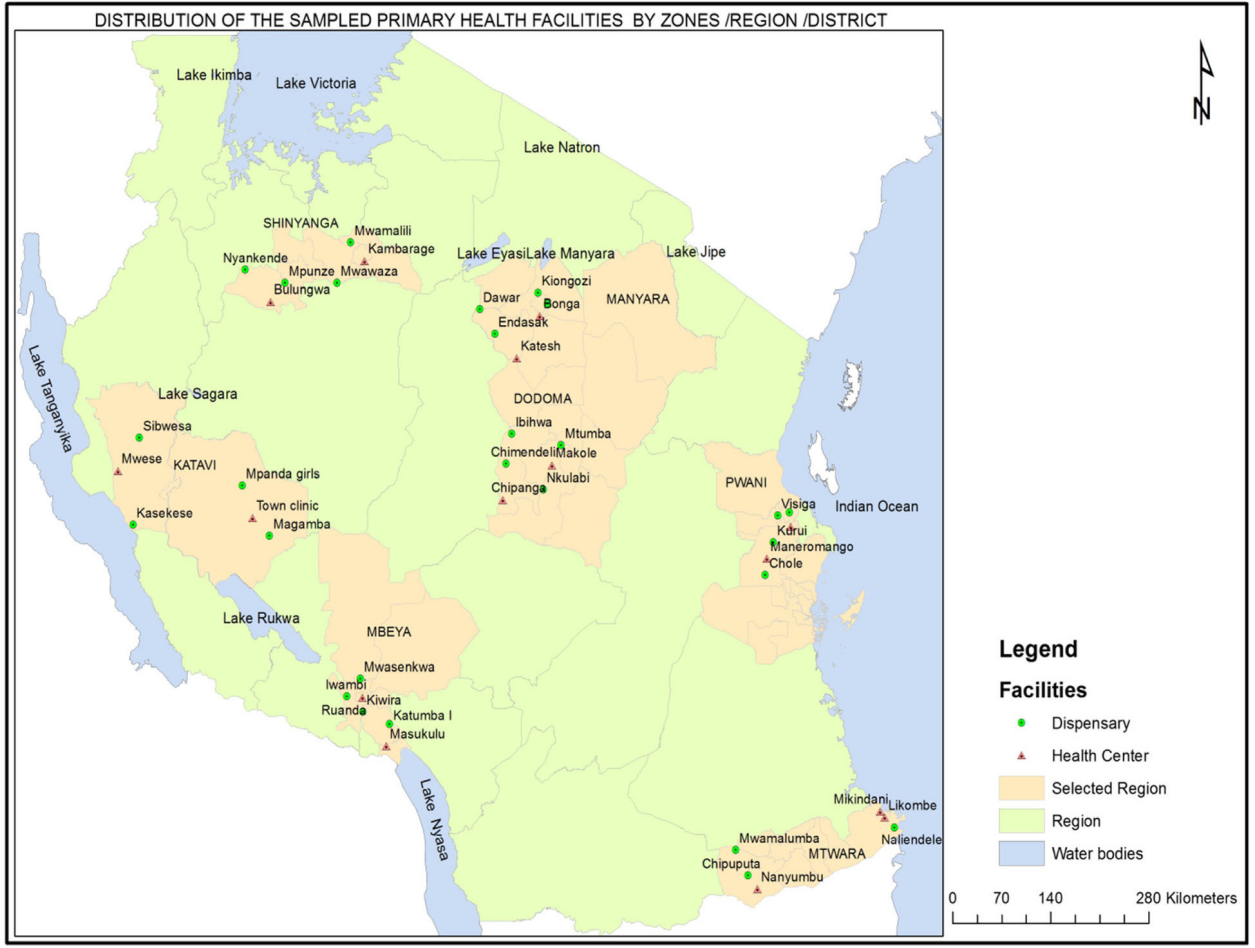
Distribution of the sampled primary health facilities by zones/regions/districts

Tanzania’s mainland is divided into 7 geographical zones. Each zone has an average of 3–4 regions. There are 26 regions in the country with 185 district councils. This study will be conducted within 14 local government councils drawn from 7 out of the 26 regions of Tanzania (Fig. 1), covering a population of 14 million (as per the 2012 census projections), representing 25% of the Tanzanian population [33].

### The DHFF programme

The implementation of the DHFF programme started from the midpoint of (February, 2018) the 2017/2018 fiscal year in all district councils in Tanzania, under the guidance of the President’s Office – Regional Administration and Local Government. The DHFF programme is a government initiative that aims to implement fiscal decentralisation in the health sector whilst fostering health services improvement [26]. The programme was introduced to meet the following goals: (1) improving the structural quality of maternal and child health services by improving the services pertaining to these two groups; (2) increasing accountability and governance in the health system at the primary healthcare level; (3) increasing health system responsiveness for patients who receive healthcare in the respective health facilities; and (4) improving health seeking behaviour and service utilisation at the primary health facility level and avoiding bypass at this level. More than 95% of the Tanzanian population live within 5 km from a primary health facility (dispensary or health centre) [34].

Prior to the DHFF programme, districts managed and controlled funds for primary healthcare facilities and, therefore, facilities had no direct access to cash or to direct control of financial resources. Some funding sources that were collected at the health facility, including user fee revenue, National Health Insurance Funds and Community Health Fund premiums, were deposited into the district account. Districts would plan for health activities and budget for these facilities each year. This resulted in delays in the implementation of various health interventions contributing to the poor quality of health services delivery. This was coupled with poor autonomy at the primary health facility levels and responsible health facility-governing committees. The DHFF programme envisages increasing motivation and autonomy of HFGC as seen in other countries, for example, in Kenya [11].

The DHFF programme implementation includes the following components: (1) training on DHFF programme, through which 138 regional team members will be trained so that they can, in turn, train others at district councils through the cascade approach; (2) supportive supervision and mentorship; (3) employment of assistant accountants; (4) dissemination of facility Financing Accounting and Reporting Systems and DHFF programme implementation guidelines; (5) opening of accounts approved by the Bank of Tanzania; (6) disbursement of funds to approved facility accounts; and (7) provision of tools for DHFF programme implementation and coordination. The Regional Health Management Team and CHMT will provide technical support and mentorship on financial management, implementation of annual plans and budget to the health facilities in accordance with the provided guidelines. It is anticipated that the programme will lead to specific changes, including an increase in the structural quality of maternal health services, an increase in accountability and governance at the primary healthcare level and an increase in health system responsiveness, ultimately leading to an increase in service utilisation and health system performance in general.

### Study design

We will employ a before and after, non-controlled, mixed methods study design to evaluate the effects of the DHFF programme on the health system and to study programme implementation. As the DHFF programme represents a nationwide reform in the manner in which health facilities are financed, controlled study designs are not feasible. Quantitative methods will be used to measure the effects of the programme, whereas qualitative methods will be used to attribute observed effects to the programme and to provide in-depth understanding of how the programme works in actual practice [35–37]. The evaluation will be informed by Theory of Change (ToC), the conceptual framework for this study.

### Theory of Change (ToC) for the DHFF programme

Developing the ToC, or a programme theory, is the first prerequisite in understanding the implementation processes and effects of any programme [38]. The ToC helps to open the black box and establish potential causal pathways between the DHFF programme inputs and the expected outcomes. Specifically, the DHFF programme, by increasing provider autonomy over access to and use of resources, is assumed to increase engagement of health facility governing committees in the planning and financing of care, and to result in the improved structural quality of care as it allows for facility resources to be directly invested in service delivery. The successful implementation of the programme relies on providers receiving training about the programme and understanding how it works, including the implementation of a new financial reporting system. Similarly, assistant accountants will be required to support the financial management of resources by providers and assist in the generation of financial reports. As providers have more autonomy over the use of resources, they should become more conscious of opportunities to raise revenue through client user fees and, as a result, become more responsive to client needs. This increased responsiveness, coupled with the improved structural quality of facilities, should result in greater utilisation of services among patients. The ToC assumes that district managers will support the new decentralised system by providing supervision and mentorship to providers and support programme implementation. However, it is equally possible that they may see the programme as a threat, removing their control over resources and limiting their oversight of resources.

**Fig. 2:**
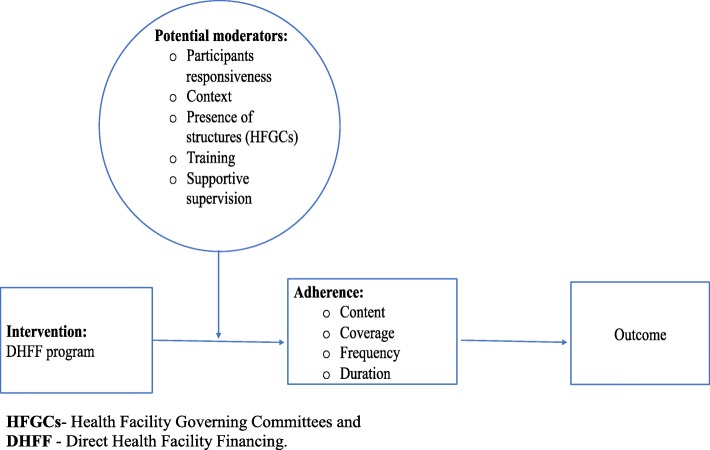
Conceptual framework for implementation fidelity (originally from Carroll et al. [38])

The study measurements of DHFF programme implementation will be guided by the Fidelity of Implementation (FoI) framework, as stipulated in some studies conducted in other countries [38, 39]. As a framework, FoI takes into consideration the issue of adherence to the original programme design and all the moderating factors. This affects adherence to complementary parts of a comprehensive approach to measuring and understanding implementation. Therefore, as part of the process evaluation study, we intend to assess some moderating factors, including training, supportive supervision, and both financial assistance and guidelines for the DHFF programme implementation. Moreover, we will study potential adherence factors through assessment of staff training and its content, implementation of supportive supervision and mentorship after training, and service providers’ responsiveness. We will also pay attention to the context of practice and scrutinise structures through which the programme was supported and implemented (Fig. 2). The moderating factors and adherence will be evaluated through observations, interviews and document reviews.

**Fig. 3:**
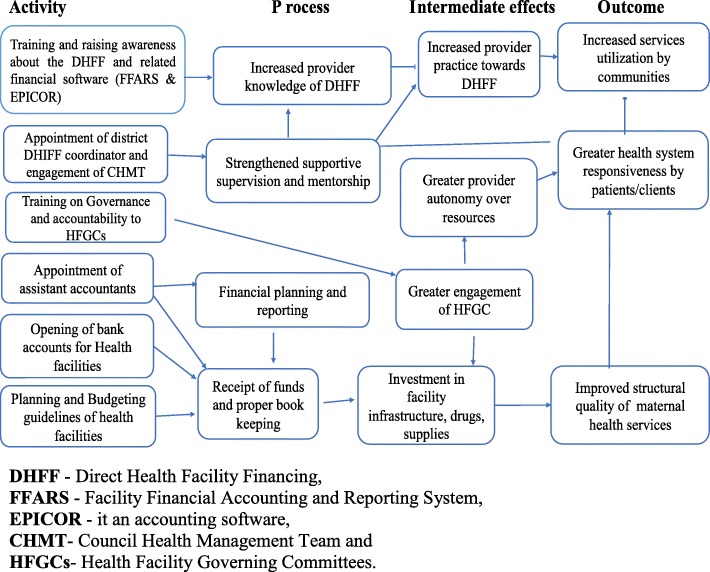
Theory of Change framework for the DHFF programme implementation in Tanzania

A ToC for DHFF was conceptualised during two stakeholder meetings, namely the Health Basket Fund sub-committee meeting on audit and performance in July 2017, whereby participants spelled out the processes of change they anticipated, and a subsequent meeting of the Health Basket Fund Committee, which set out the necessary steps in DHFF programme implementation. The authors of this paper further refined the ToC to be utilised in the proposed evaluation, based on a review of the literature (Fig. 3).

### The study sample

The participants in this study will be drawn from the seven regions of Tanzania. The seven regions were selected at random from each of the seven geographical zones. Two districts were then selected at random from each of the seven regions. The 14 district councils contains approximately 525 health facilities, i.e. 8.5% of all health facilities in the country. The average healthcare seeking behaviour and maternal mortality is comparable to all other district councils in the country [33].

### Sample for measuring health system effects

A total of three primary health facilities were selected through stratified sampling then randomly drawn from each district’s list of types of public primary healthcare facilities (i.e. health centres, dispensaries) (https://hfr-portal.ucchosting.co.tz/index.php?r=facilities/homeAdvancedSearch) [40], making a total of 42 health facilities (14 health centres and 28 dispensaries).

From each selected health facility, the staff member in-charge and the HFGC chairperson will purposefully be selected, while exiting patients will be systematically selected after gender stratification following medical consultations. The exit interview patients will be approached after they have received the services and are ready to go home. Respondents eligible for interview include all exiting patients or relatives of patients (aged above 18 years). They will be sampled to ensure equal numbers of men and women are captured. A total of 422 patients will take part in exit interviews. The sample size was calculated using the Cochran formula (1977) [41]; by taking 50% as a proportion of patients’ perception to health system responsiveness (as there are no previous similar studies performed in Tanzania) and a power of 80% allowing for an estimated error margin of 5%, the sample size obtained was 384 patients. An additional 10% of the sample size was added (*n* = 38) to allow for refusals, making a total of 422 patients.

### Sample for the process evaluation

Our sample for the process evaluation will include DHFF programme implementers (health workers, members of HFGCs, CHMT members) and patients exiting health facilities from each participating primary healthcare facility. Employing a mixture of qualitative and quantitative tools is important to explore the process of implementation and to appreciate the contexts of the intervention in order for the effects to be interpreted appropriately. The tools will be made in light of the ToC and the FoI conceptual framework (Additional file 1).

In conducting a process evaluation, the ToC (Fig. 3) will be used as a guide to measure the implementation processes by assessing each input, namely training, supportive supervision, mentorship, the presence of DHFF coordinators and the opening of a facility account with the Bank of Tanzania, together with its effect on structural quality, health system responsiveness, service utilisation and health system performance.

Another conceptual framework which will guide the the process evaluation is the FoI (Fig. 2), in which the moderating factors, such as training on DHFF, guidelines and policy guidance for the implementation and the adherence to the programme implementation, will also be assessed.

### Data sources

The sources of data for this study are grouped according to the two functions this study aims to achieve, namely assessment of health system effects and assessment of the implementation process. We provide a description on the data sources for each function.

### Health system effects

The health system effects of the interventions will be measured at baseline, midpoint between February and May 2018, and 18 months afterwards (ending in August, 2019) in all 42 facilities using four survey tools, through surveys of health facility in-charges and chairpersons of health facility governing committees and exit interviews with patients accessing healthcare services. A facility observation checklist will also be used (Additional file 2). Data on service utilisation for the previous 12 months will also be extracted from the District Health Information System version 2 platform or the web portal (https://hmisportal.moh.go.tz/hmisportal/#/) [42].

#### Survey of in-charges

The survey of facility in-charges will capture information related to the structural quality of maternal health services (e.g. labour services, hygiene and sanitation, medical equipment, staffing levels and service utilisation, infection prevention in addition to maternal death audits) (Additional file 2). The questions in this tool were drawn from the national Results-Based Financing programme selected quality indicators for maternal and child health services [43, 44] combined with the WHO framework to oversee standards of care to improve maternal and newborn quality in the health facilities [45].

#### Observation checklist

A structured observation checklist will be used to collect data on the structural quality of maternal health services. This will serve as triangulation of the information obtained from the survey of facility in-charges.

#### Health Facility Governing Committee (HFGC) questionnaire

This questionnaire will capture information related to the role of the HFGC in relation to the implementation of the DHFF programme and the management of resources, and the involvement of other stakeholders in this process, in addition to the effect of the programme on relationships between different levels of the health system and stakeholders at the primary care level. It will look at the governance and accountability of these committees in all primary health facilities and to the community.

#### Patient questionnaire

An exit interview will be administered to 10 patients per health facility to measure patient experiences with healthcare in relation to prompt attention, access to care, respect of dignity, quality of communications, quality of basic amenities, confidentiality and autonomy. We will use a 37-item questionnaire adapted from the health systems responsiveness questionnaires used in WHO multi-country studies [46]. Items will be measured using 3- or 4-point Likert scales. To ensure reliability of the tools, the internal consistency of the overall scale (37 items) will be measured using Cronbach’s alpha [47].

### Process evaluation

The process evaluation will be conducted 6 months after the national DHFF programme implementation in public primary health facilities. The second round will be repeated 18 months after the baseline study.

#### Structured questionnaire for implementers

We will administer structured questionnaires to programme implementers (health facility in-charges, district medical officers and health facility governing committees, and health service providers) to capture their knowledge of DHFF, FoI and the factors influencing/moderating FoI, for example, training, policy and guidelines, and supportive supervision and mentorship. We have developed each distinct questionnaire to reflect the specific type of the implementation team being interviewed. Confirmatory factor analysis will be completed, followed by a test of reliability of the tools using Cronbach’s alpha (which needs to be 0.7 or above). All the tools will be pre-tested before actual data collection.

#### In-depth interviews

We will undertake in-depth interviews to aid understanding of the implementation processes and factors influencing it, as well as accountability and governance of DHFF programme, and changes to existing structures brought about by the programme. The individuals will be selected based on in-depth understanding of the subject matter (Table 2).

#### Focus group discussions (FGDs)

FGDs will also be conducted with health service providers. The FGDs will be used to assist in gaining a better understanding of the DHFF programme. A total of 12 FGDs will be conducted, although reaching saturation will decide the amount of data to be collected. These FGDs are important to gain an understanding of the views of health service providers on the DHFF programme.

### Data collection procedures

#### Survey data

Health worker and patient data will be captured using mobile devices on a daily basis. To ensure accuracy of the collected information, research assistants will undergo 4 days training on survey data collection using paper-based tools and mobile devices (Samsung Galaxy Tablets 7.0) prior to taking part in pre-testing the tools. There will be a web-based interface that allows real-time gathering of data from selected health facilities. All selected facilities will have GPS coordinates and all the data enumerators will use tablets with GPS sensors. The lead author will monitor data collection on a daily basis to ensure the integrity and quality of data. Each day, data will be sent directly to the Gmail account app (which will act as a server) after being filtered in the field. The data collected via the mobile phone will be uploaded using data collection software with skip and quality check functions to minimise data entry error. Data will be transferred into an Excel sheet and converted to SPSS version 22 for analysis. Hard copies of questionnaires will be stored in a locked room. The survey and observation data will be captured on paper and double-entered into a pre-designed database.

#### Qualitative data

Interviews and FGDs, conducted as part of the process evaluations, will be conducted in Kiswahili by trained research assistants and recorded using audio digital recorders. We will use semi-structured interview topic guides to collect data from participants during in-depth interviews and FGDs. Audio files will be transcribed by the research assistants who conducted the interviews by using F4 programme and then will be translated into English by the bilingual researcher who will also conduct the interviews. All transcripts will be imported into QSR NVivo 8 for data management.

### Data analysis

#### Quantitative data analysis

Analysing quantitative data requires an understanding of the measurements of each of the variables in question and the use of statistical approaches to either describe the data or to find relationships between the variables. On that regard, the data management plan was prepared for both outcome and process evaluation to guide the data analysis exercise (Tables 1 and 2).

**Table 1:** Data management for outcome evaluation objectives

Specific objectives	Variables	Tools for data collection	Analysis technique
Objective one	Structural quality of maternal health	7 items observational checklist with a total score of 79 12 items observational checklist which assess number of attendances by patients who seek different health services (Service Utilisation)	Descriptive statistics (mean, standard deviation) Cross tabulation to compare groups’ performance Regression analysis for checking statistical significances among and between the participants Paired sample *t* test for comparing means between the baseline and end-line surveys
Objective two	Governance and accountability	In-depth interview guide	Thematic analysis
Objective three	Health system responsiveness	37 questionnaire items divided into four groups: Ordinal variables range 0 (never) to 3 (very often) Ordinal variable ranging from 0 (very big problem) to 3 (no problem) Ordinal variable ranging from 1 (waited for long time) to 4 (serviced instantly) Ordinal ranging from 1 (strongly disagree) to 4 (strongly agree)	Descriptive statistics frequency of scores distribution and mean and standard deviation Cross tabulation for checking differences of scores among the participants Regression analysis for checking associations among the variables

**Table 2:** Data management for process evaluation objective

Specific objectives	Variables	Measurement	Analysis technique
Objective one on process evaluation	Knowledge on Direct Health Facility Financing (DHFF) programme	24 questionnaire items with categorical variables	Descriptive statistics on frequency, mean and standard deviation Cross tabulation to compare performance of the participants
	Practice of DHFF programme in relation to Fidelity of Implementation framework	14 questionnaire items with categorical variables 10 observational checklist items of financial management at the facility	Descriptive statistics on frequency, mean and standard deviation Cross tabulation to compare performance of the participants
	Acceptability to DHFF programme	20 semi-structured interview questions	Thematic analysis

### Study outcomes

#### Measuring health system effects

The before and after study will provide quantitative measures of a number of health system outcomes related to the ToC (Fig. 3), including health system responsiveness. This measures the non-health aspect of care relating to the environment and the manner in which healthcare services are provided to clients across seven domains; specifically, attention is defined as care provided readily or as soon as is necessary [36], autonomy is defined as a freedom of the will [36], amenity of care is related to the extent to which the physical infrastructure of a health facility is welcoming and pleasant [36], access to care is entry into or use of the healthcare system [36], communication is defined as the clarity in conveying information and evoking understanding [36], respect for dignity is defined as the state of being worthy of honour or respect [36], and confidentiality is defined as being entrusted with secrets and non-exposure of the body to other people [35, 36]. Health system responsiveness will be measured through mean scores of the items contained in each of the seven domains of health system responsiveness [48].

##### Structural quality of maternal health services

Structural quality of maternal health services will be measured using mean scores for each item of the structural quality indicators as stipulated in the observational checklist. Structural quality is obtained through assessment of the characteristics of a care setting, including facilities, personnel, and/or policies related to care delivery.

##### Governance and accountability

Information will be obtained through in-depth interviews and FGDs about supportive supervision, HFGC meetings, and their roles and responsibilities. This information will then be analysed by means of thematic analysis.

Table 1 provides the variables for the process evaluation study where the dependent variables are health system performance and service utilisation and the independent variables are knowledge, acceptability and fidelity of implementation.

##### Service utilisation

This study will also look into service utilisation using general and maternal health-related indicators such as attendance at antenatal clinics, outpatient department attendance, institutional deliveries, intermittent presumptive treatment for malaria, and postnatal care. The source of information for service utilisation will be the District Health Information System version 2. The veracity of information will be measured by checking through the monthly submission forms and respective Health Management Information System books/registers, i.e. Health Management Information System Book/Register number 5 (outpatient department attendance), Book number 6 (antenatal care visits, intermittent presumptive treatment for malaria) and Book number 12 (institutional delivery).

We will also generate a composite health system performance outcome, which combines all items captured under health system responsiveness, structural quality of maternal health services and service utilisation into a single composite index. The health system performance will be categorised into two groups, namely good and poor health system performance, using data obtained from the individual surveys. As an estimation method, a composite index will be used to assess the overall weighted average of the three variables of health system responsiveness, structural quality of maternal health services and service utilisation, in accordance with the approach used by WHO [3]. To examine how health system effects vary by context, we will also document the characteristics of facilities, providers and patients.

### Measuring implementation

#### Fidelity of Implementation (FoI)

We will use several questions to determine whether the DHFF programme is being implemented according to the design and to identify areas of deviation. The mean score will be used to decide whether the health service providers demonstrated high or low fidelity of implementation to the DHFF.

We will also examine the effect of DHFF programme activities on health workers, for example, the training provided to them, the number of participants who attended, their level of awareness and knowledge of the programme, the number of assistant accountants recruited and timeliness of funds transferred from the treasurer. A total of 20 questions related to items carried out during DHFF programme implementation will be asked of providers and a mean score computed across all 20 items. A mean score for the questions will be computed. Those who scored above the mean value will be graded as having adequate knowledge, while those scoring below the mean score will be categorised as having inadequate knowledge. The categories will be across zones, level of health facilities and management level. Multiple correspondence analysis will be performed in order to generate scores.

#### Acceptability by health service providers

The health service providers involved with DHFF programme implementation will analyse this based upon the themes emerging from the in-depth interviews.

#### Facilitation strategies (moderating factors)

In this study, there will be an assessment related to the complexity of the programme and understanding the health services providers’ perceptions of the DHFF programme implementation. The facilitation strategies to address complexity and perception will be assessed by using a questionnaire. Factors to be studied are training, supportive supervision, mentorship, and participants or health service providers’ responsiveness. Regarding the adherence section, we will study the context factors (i.e. presence of an active HFGC, having been trained on DHFF programme implementation, the type of facility (whether it is a dispensary or health centre), staffing level, location of the facility (i.e. urban or rural), dose, and the coverage of DHFF programme (Fig. 2). We will also describe all these factors as per the FoI conceptual framework [38].

### Statistical analyses

Tests of differences in health system outcome means between baseline and end-line surveys will be conducted using the paired sample *t* test. We will run a multiple logistic regression model (as outcome variables will be grouped into two categories and there is more than one covariate) for each health system outcome at a time.

### Qualitative data analysis

All the interviews will be audio-recorded by the digital tape recorders then transcribed through the use of the F4 programme and saved into a computer-based text file.

Transcripts will then be imported to NVivo version 12 (QSR International Pty Ltd., Australia) for coding and sorting. Transcripts will initially be examined to identify primary coding categories within themes. Codes and sub-codes will be derived directly from the transcripts. A codebook containing identified coding categories and newly emerging themes will be developed and attached in the appropriate code as coding proceeds. The purpose of this process is to systematically group text data into fewer content-related themes that share the same meaning. A thematic content analysis approach guided by use of NVivo 12 software will be employed.

## Discussion

Understanding the implementation of the DHFF programme and its effects on the health system is crucial to gaining insights into how such a fiscal decentralisation policy interacts with the health system. Evolution of this complex intervention and subsequent pathways to influence change in the healthcare system will depend both upon how the programme components are implemented and how the programme interacts with the context. Gaining an understanding of the programme implementation and the contextual interactions provides an important step in discovering what effects the programme will produce on health system outputs (responsiveness, quality and accountability). Therefore, through the evolution of this DHFF programme, it is expected that improvement in the health-seeking behaviour and health service utilisation for people at the community level will result by initial utilisation of the primary healthcare service prior to progressing to secondary and tertiary service provision levels, helping to reduce bypassing. This is important in Tanzania as the majority (95%) of health facilities in the country are at primary healthcare level and are the first entry point to the healthcare system. These facilities are close to the communities [34]. Therefore, this intervention is expected to strengthen not only the healthcare system performance but also the referral system.

Although the Tanzanian health system has experienced fiscal decentralisation reforms, mainly regarding locally collected revenue, the current DHFF programme includes central government and donor funds and may therefore attract interest from various stakeholders. The most important aspect has been the fact that, through the Health Basket Fund committees and sub-committees, there has been buy-in from stakeholders on this approach, and it is therefore very important to see how the implementation evolves. The financial autonomy brought to primary healthcare facilities and their governance structures by these reforms may impact decision space and therefore influence the health system outputs.

This study, through both process and effect (impact) evaluation, is expected to uncover knowledge gaps that require rectification for the effective implementation of the DHFF programme, and will inform policy and decision-makers as to the issues of concern, while helping with the development of ToC. Evaluation of the study findings will also be of significance to researchers and policy-makers in the field of health financing and health systems as a whole. Understanding how the DHFF programme implementation has been affected either positively or negatively will aid in taking appropriate further measures. The ToC pathways will aid policy-makers and implementers in deciding how and where to intervene, should things not be progressing in the right direction.

The main limitation of this study is the before and after design, which is non-controlled, as the DHFF programme will be implemented as part of a nationwide programme rollout. A case control study or controlled quasi-experimental design was not possible as implementation started simultaneously across all regions. To ensure that the observed changes are attributed to the programme, assessment of implementation outcomes and documentation of contextual factors will be undertaken. Strategies to understand contextual changes include the establishment of a surveillance system with special arrangements to track any incoming events, projects, programme or any support in the study areas. This will help in understanding any external contributing factors to the studied objectives and indicators. Moreover, some questions in the tools have been set to assess the progress of the approach and trace any new programme that might be introduced along with the study programme. Secondly, there will be triangulation through the use of different data collection methods in which different responses will be verified as well as the use of more than one data collection tool.

Other constraints include the fact that impact evaluation will be performed within 18 months whereas process evaluation, which explores changes, will be completed within 1 year. These two different time frames are relatively short and, therefore, other studies are required to explore sustainability issues.

## Conclusion

This study protocol is important because it lays down the grounding for subsequent studies to be conducted. It enables the impact and process evaluations to be conducted with a high level of precision. It is envisaged that this protocol will be used as reference material for evaluation studies in areas related to DHFF as a key component of health system performance as we move towards universal health coverage.

## Additional files

Additional file 1: Tools for assessing the implementation of the DHFF programme (process evaluation) (English/Kiswahili). (DOC 248 kb)

Additional file 2: Tools for assessing the effects of the DHFF programme (English/Kiswahili). (DOC 376 kb)

## Abbreviations

CHMT: Council Health Management Teams
FGD: Focus Group Discussion
FoI: Fidelity of Implementation
HFGC: Health Facility Governing Committee
RCH: Reproductive and child health
ToC: Theory of Change
