# Supplementary material for: Understanding the implementation of Direct Health Facility Financing and its effect on health system performance in Tanzania: a non-controlled before and after mixed method study protocol
Source: Health Res Policy Syst. 2019 Jan 30;17:11. doi: 10.1186/s12961-018-0400-3 (PMC6354343; doi:10.1186/s12961-018-0400-3)
Supplement: Supplementary file 1 — Tools for assessing the implementation of the DHFF programme (process evaluation) (English/Kiswahili). (DOC 248 kb) [file 12961_2018_400_MOESM1_ESM.doc]

**Additional file 1: Tools for assessing the implementation of the DHFF program (process evaluation) (English/ *Kiswahili*)**

**(a) In-depth Interview guide for Chairman of HFGCs**

***(a) Mwongozo wa hojaji la wenyekiti wa kamati ya usimamizi wa kituo cha afya.***

***Utekelezaji wa program ya upelekaji wa fedha za huduma ya afya moja kwa moja kwenye vituo vya afya nchini Tanzania: Hojaji la tathmini ya mchakato.***

**Objectives**

- Assess DHFF implementation progress
- Generate and investigate hypotheses on individual-level changes and changes in relationships between actors/levels (District and Facility Level) from DHFF
- Document contextual factors that might affect intervention impact

**Icebreaker**

1. How long have you been working as a chair of HFGC in this facility?

***Ni kwa muda gani umefanya kazi kama Mwenyekiti wa wa kamati ya usimamizi wa kituo***

1. What are your roles as a Chair to the HFGCs? What roles do you provide?

***Kama Mwenyekiti wa kamati ya usimamizi majukumu yako ni yapi ? na unatekeleza majukumu gani?***

1. Do you have the necessary skills to carry out the roles described above (question 2)? Please specify for which role you have the necessary skills and for which roles you do not have the necessary skills.

***Una utalaamu wa msingi juu ya nanmna ya kutekeleza majukumu uliyoainisha hapo juu? Taja ni utaalamu gani ulionao na usiokuwa nao katika utekelezaji wa majukumu yako.***

1. What do you think a health committee should be doing (in addition to the roles described

in 2)?

***Je , Unadhani ni kitu gani cha ziada ambacho Kamati ya usimamizi wa kituo ingefanya zaidi ya majukumu uliyoainisha hapo juu?***

1. The guidelines for health committees’ work says that health committees should carry out the following tasks (listed as A,B,C,D):

***Muongozo wa kamati ya usimamaizi ya kituo unaitaka kamati kutekeleza kazi zifuatazo (kama zilivyorodhehswa kwenywe vipengele A, B, C na D)***

1. “Provide governance as it relates to service provision within the facility/facilities”

***Kusimamia masuala ya utawala na uongozin katika kituo kulingana na miongozo ya utoaji wa huduma .***

1. How do you understand this task? Please describe in your own words:

***unalitekelezaje jukumu hili? Tafadhali elezea.***

1. Do you have the necessary skills to carry out this task? a)Yes b) No

***Je, umepata mafunzo yoyote ya kukuwezesha kutekeleza jukumu hili a)Ndio b) Hapana***

1. “Take steps to ensure that the needs, concerns and complaints of patients and the community are properly addressed by the management of the facility”

***Kuchukua hatua na kuhakikisha kuwa mahitaji, matakwa na malalamiko ya wagonjwa na jamii yanafanyiwa kazi ipasavyo na menejimenti ya kituo.***

1. How do you understand this task? Please describe in your own words:
2. ***je, unatekelezaje jukumu hili? Tafadhali eleza***

1. Do you have the necessary skills to carry out this task? a)Yes b)No c)N/A

***Je, umepatiwa mafunzo yoyote ya utekelezaji wa jukumu hili? a)Ndio b) Hapana***

1. “Foster community support for the initiatives and programmes of the facility”

***Kuhamasisha ushiriki wa jjamii katika miradi ya maendeleo ya kituo.***

1. How do you understand this task? Please describe in your own words:
2. ***Je, unatekelezaje jukumu hili? Tafadhali eleza***
3. Do you have the necessary skills to carry out this task? a)Yes b)No c)N/A

***Je, umepatiwa mafunzo yoyote ya utekelezaji wa jukumu hili?a)Ndio b) Hapana***

1. “Monitor the performance, effectiveness and efficiency of the facility/facilities”

***Kufuatilia utendaji, ufanisi na ubora wa kituo.***

1. How do you understand this task? Please describe in your own words.
2. ***je, unatekelezaje jukumu hili? Tafadhali elezea***
3. Do you have the necessary skills to carry out this task a)Yes b)No c)N/A

***Je, umepatiwa mafunzo yoyote ya utekelezaji wa jukumu hili?a)Ndio b) Hapana***

1. Please list the training that you have attended as part of a health committee in the following table:

***Tafadhali orodhesha idadi na aina ya mafunzo uliyowahi kupata katika nafasi yako ya mwenyeketi wa kamati ya usimamizi wa kituo***

| Training Course  ***Aina ya mafunzo*** | How long was the course?  ***Muda wa mafunzo*** | Who offered the course?  ***Aliyetoa mafunzo*** | Did you receive a certificate? (Yes or No)  ***Je ulipata cheti? (Ndio au Hapana)*** | How useful was the training?  (Refer to the key)  Mafunzo yalikuwa na manufaa kwa kiasi gani?  (Rejea ufunguo) | Please describe briefly why it was useful or not?  ***Eleza kwa kifupi namna gani yalikuwa na umuhimu au la*** |
| --- | --- | --- | --- | --- | --- |
|  |  |  |  |  |  |
|  |  |  |  |  |  |
|  |  |  |  |  |  |
|  |  |  |  |  |  |
|  |  |  |  |  |  |

Key/ Ufunguo:

1. Not useful/ Hayakuwa na manufaa
2. Slightly useful/ Yalikuwa na manufaa kiasi
3. Very useful/ Yalikuwa na manufaa sana
4. Which training course/s was/were the most useful you have received whilst a Health Committee member that you feel you are currently using in your role as a committee member?

***Ni mafunzo gani ambayo ulipatiwa katika kipindi chako cha kuwa mwenyekiti wa kamati ambayo unayatumia zaidi katika kutekeleza majukumu yako kwa sasa?***

1. What previous experiences have provided you with skills useful to be a health committee member and chairperson?

***Ni uzoefu gani ulio nao wa awali ambao umekupatia ujuzi wa kuwa mjumbe na mwenyekiti wa kamati ya usimamizi wa kituo hiki?***

1. When you joined the health committee, did you receive any orientation or induction? Please explain.

***Je, ulipochaguliwa kuwa mjumbe wa kamati ya usimamizi wa kituo, ulipatiwa mafunzo yoyote au maelezo ya utangulizi kabla ya kuanza utekelezaji wa majukumu yako? Tafadhali eleza.***

1. What training do you think an orientation and induction programme for new health committee members should include?

***Unadhani mafunzo gani au maelezo gani ya utangulizi ambayo mjumbe wa kamati ya usimamizi wa kituo anatakiwa apatiwe ?***

1. Have you been offered any training that you were unable to attend? A) Yes b) No

***Ulishawahi kupatiwa fursa ya mafunzo na ukashindwa kuhudhuria? A) Ndio b)Hapana.***

1. If you answered yes to question 12: what was the reason for not being able to attend the

training?

***Kama umejibu ndio kwenye swali la 11; ni sababu zipi zilikufanya ushindwe kuhudhuria mafunzo hayo?***

1. What are the roles for financial resources management for you as a chair of HFGC?

***Ni yapi majumu yako ya usimamizi wa fedha kama mwenyekiti wa kamati ya usimamizi wa kituo?***

1. Is there any challenges you are facing in the course of financial management practices?

***Je kuna changamoto gani ambazo unakumbana nazo kwenye usimamizi wa fedha?***

**Mechanisms of impact**

1. What is your relationship with health facility staff in management of health facility fund?

***Elezea uzoefu wa mahusiano kati yako na watumishi wa kituo katika usimamizi wa fedha za kituo?***

1. What is your relationship with CHMT members in the management health facility fund ?

***Elezea uzoefu wa mahusiano kati yako na timu ya usimamizi wa afya ya wilaya kwenye usimamizi wa fedha za kituo?***

1. What is your relationship with community in relation to facility management?

***Elezea uzoefu wa mahusiano yako na jamii katika usimamizi wa kituo?***

1. Has your role as chair of HFGC affected any of your routine activities? If so, how? How do you handle this?

***Katika majukumu yako kama mwenyekiti wa kituo yameathiri vipi kazi zako za kila siku?***

1. What is your convenience in the availability and management of resources at the facility level?

***Upi uzoefu wako katika upatikanaji na usimamizi wa rasilimali katika katika ngazi ya kituo.***

- 1. Drug supply management

***Usimamizi wa usambazaji wa dawa***

- 1. Equipment

***Vifaa tiba (mf. Vitanda, mizani nk)***

- 1. Staffing

***Watumishi***

1. What are the different projects are being implemented in this health facility?

***Ni miradi ipi ya maendeleo inayotekelzwa katika kituo hiki cha afya? (Mf. Mradi wa mama na mtoto, Malaria, UKIMWI na Kifua Kikuu)***

1. Are there any social economical factors that contribute or affect project implementation here?

Probes: cultural issues, political and policies.

***Je, kuna sababu zozote za kijamii, kisiasa na kiuchumi zinazochangia au kuathiri utekelezaji wa miradi hapa?***

***Chunguza; sababu za kitamaduni, kisiasana za kisera.***

1. In your experience, how can these challenges be addressed?

***Kwa uzoefu wako, ni njia gani zinaweza kutumika kutatua changamoto hizo?***

1. Do you have a Village Health Committee?

***Je mnakamati ya afya ya kijiji?***

1. If yes, how do you work with them?

***Kama ndio, mnashirikianaje katika kazi?***

1. Do you get remunerated with any incentives? If yes, What type?

***Je, huwa unapewa motisha yoyote katika kuyatekeleza majukumu yako? Kama ndio, ya aina gani?***

# (b) Questionnaire for DHFF program implementers.

***(b) Dodoso la watekelezaji wa mradi***

**Health service providers**

***Watoa huduma za Afya***

**Objective No.1;** To asses the Knowledge, Acceptability and Practice of Heath service providers towards DHFF program implémentation

Serial No. **[__|__|__|__]**

Name of the Region: _____________________________________

***Jina la Mkoa***

Name of the District Council: ______________________________

***Jina la Wilaya*** [_____________]

Name of the Health Facility: _______________________________

***Jina la kituo cha kutolea huduma***

Type of Health Facility: **01**= Dispensary/*Zahanati* **[ ] 02=** Health center/ *Kituo cha Afya* **[ ]**

***Aina ya Kituo*** [________]

**Please put the appropriate number of a response in the given *box.***

***Tafadhali jaza namba yenye jibu/majibu sahihi***

PART 1: HEALTH SERVICE PROVIDERS

Sehemu ya kwanza:watoa Huduma

| **SN** | **Questions/*Maswali*** | | **Responses /*Majibu*** | **Code** | **Code** |
| --- | --- | --- | --- | --- | --- |
| **SECTION A: DEMOGRAPHIC INFORMATION** | | | | | |
|  | (a)Sex/***Jinsi*** | 1.Male/***Mwanaume***  2.Female/ ***Mwanamke*** | | 01  02 | [ ] |
|  | How old are you?  ***Una umri gani?*** |  | |  |  |
|  | Marital status  ***Hali ya ndoa*** | 1.Married/***Nimeoa/Nimeolewa***  2.Cohabiting/***Tunaishi pamoja bila ndoa***  3.Single/ **Sijaoa/Sijaolewa**  4.Divorced/Separated/***Tumeachana***  5.Widow/widowed/***Mjane/Mgane*** | | 01  02  03  04  05 | [ ] |
|  | Highest level of education  ***Elimu yako*** | 1.Certificate/***cheti***  2.Diploma/***stashahada***  3.Advanced diploma/***stashahada ya juu***  4.University degree/ ***shahada***  5.Masters (MMED)/ ***shahada ya uzamili***  6. Masters (MPH)/***shahada y***a ***uzamivu ya utawala wa afya ya jamii***  7. Masters (MSc) *shahada ya uzamili ya sayansi*  8. Other (Mention)…..  ***Nyingine (Taja)……..*** | | 01  02  03  04  05  07  08 | [ ] |
|  | What is your cadre?  ***Taalum uliyosomea*** | 1.Enrolled Nurse  ***Nesi mwenye cheti***  2.Registered Nurse  ***Nesi mwenye astashahda***  3.Nurse Officer  ***Nesi mwenye shahada***  4.Assistant Clinical Officer  ***Tabibu msaidizi***  5.Clinical Officer  ***Tabibu***  6.Assistant Medical Officer  ***Msaidizi wa Daktari***  7.Medical Officer/Dental Officer  ***Daktari***  8.Medical specialist (MMED)  ***Daktari bingwa***  ***9.***Assistant Accountant  ***Mhasibu Msaidizi***  10. Health Facility Governance Committee Chair/ Member  ***Mwenyekiti wa Kamati ya Usimamizi wa Kituo.*** | | 01  02  03  04  05  06  07  08  09  10 | [ ] |
|  | How long have you been working in your current job/position? [Clinician]/ [nurse] *(record number of years or term served)*  ***Je! umefanya kazi kwa muda gani?*** |  | |  | [ ] |
|  | What is your position at this health facility?  ***Unacheo gani katika kituo chako cha kazi?*** | 1. Health service provider 2. Matron 3. In charge 4. Assistant Account 5. HFGC Chair/ Member. 6. Other (Mention)___________ | | 01  02  03  04  05  06 | [ ] |
|  | **Assessment of Acceptability of DHFF Program** | | | |  |
|  | In general, do you like the way DHFF program operates? *(If the answer is no skip the following questions 9-19, 27, 28, 42 – 44, 46, and 49)* | 1. No  2. Yes | | 01  02 | [ ] |
|  | How do you feel about the introduction of DHFF program? | 1.Uncomfortable  2. Comfortable | | 01  02 | [ ] |
|  | Do you agree that, DHFF is beneficial? | 1. No  2. Yes | | 01  02 | [ ] |
|  | If the answer is NO, pick the reasons from the listprovided. | 1. Work load 2. Skills on financial management 3. Unavailability of working guidelines and tools | |  |  |
|  | In the course of introduction of DHFF, were you worried about challenge associated with increase of work load? | 1. No  2. Yes | | 01  02 | [ ] |
|  | In the course of introduction of DHFF, were you worried about challenge associated with competency in running the program properly? | 1. No  2. Yes | | 01  02 | [ ] |
|  | In the course of introduction of DHFF, were you worried about challenges associated with availability of working tools? | 1. No  2. Yes | | 01  02 | [ ] |
|  | In the course of introduction of DHFF were you worried about challenges associated with reporting process? | 1. No  2. Yes | | 01  02 | [ ] |
|  | **Knowledge Assesssment** | | | |  |
|  | Have you ever heard about DHFF program?  ***Umeawhi kusikia kuhusu mpango wa kupeleka fedha moja kwa moja katika kituo cha kutolea huduma (DHFF)?*** | 1. No / ***Hapana*** 2. Yes / ***Ndio*** | | 01  02 | [ ] |
|  | Where did you heard about DHFF program?  ***Uliskia kuhusu mpango huu wapi?*** | 1. On the training  ***Kwenye mafunzo***  2. From In charge of HF  ***Kwa mfawidhi wa Kituo***  3. From other staffs.  ***Kutoka kwa watumishi wengine.***  4. Somewhere else (mention)……………..  ***Mahali pengine (Taja)……..*** | | 01  02  03  04 |  |
|  | What minimum requirements do you have for the implementation of DHFF at facilities? *(multiple selection)*  *Assess the performanc of the given variable, if the facility missed one of them then its lack minimum requirements.*  ***Je, Ni mahitaji gani ya msingi mliyo nayo kwa ajili ya utekelzaji wa mpango wa kupeleka fedha moja kwa moja katika vituo vya kutolea huduma (DHFF).*** | 1. Health facility accounts   ***Akaunti ya wa kituo***   1. At least one skilled personnel   ***Angalau mtaalam mmoja***   1. Active HFGC   ***Kuwepo kwa kamati ya usimamizi wa kituo inayofanya kazi.***   1. Others (mention)________   ***Nyingine (taja)….*** | |  |  |
|  | (FoI) Were you trained on FFARS and DHFF program  ***Umawahi kupata mafunzo ya mfumo wa Usimamizi wa fedha na kutolea taarifa wa za vituo (FFARS) pamoja n***a  ***mpango wa kupeleka fedha moja kwa moja katika kituo cha kutolea huduma (DHFF)?*** | 1. No **/ *Hpana*** 2. Yes / ***Ndio*** | | 01  02 | [ ] |
|  | **Assessment of Practice of DHFF Program** | | | |  |
|  | (FoI) Do you have active HFGC? (up to date list of members)  ***Mna kamati hai ya usimamizi ya kituo?*** | 1. No/***Hapana*** 2. Yes/**N*ndio*** 3. I don’t know **/ Sijui** | | 1  0  2 | [ ] |
|  | Do you have HFGC working guide?  ***Una muongozo wa wa namna ya kufanya kazi kwenye kamati ya usimamizi?*** | 1. No 2. Yes 3. I don’t know **/ Sijui** | | 1  0  2 |  |
|  | Do you convene quarterly HFGC meetings?  ***Je? Mnafanya vikao vya robo vya kamati ya usimamizi wa kituo?*** | 1. No /Hapana 2. Yes / Ndio 3. I don’t know **/ Sijui** | | 1  0  2 | [ ] |
|  | Do you keep minutes of HFGC meetings.  ***Mnatunza miutahsari ya vikao vya kamati ya usimamizi wa kituo?*** | 1. No/ ***Hapana*** 2. Yes (Please show)/***Ndio (angalia kuhakiki)*** 3. I don’t know **/ Sijui** | | 1  0  2 |  |
|  | Does your facility have a MoFP approved account?  ***Kituo chenu kina akaunti iliyothibitishwa na Wizara ya Fedha?*** | 1. No /***Hapana*** 2. Yes / ***Ndio*** 3. I don’t know **/ Sijui** | | 01  0  2 | [ ] |
|  | (FoI) Do you have an assistant accountant in this HF?/ Do you receive satellite accounting service?  ***Mna muhasibu msaidizi wa kituo?/ Mpata Huduma ya msaada wa masuala ya fedha toka kwa mhasibu wa kituo kingine?*** | 1. No /***Ndio*** 2. Yes /***Hapana*** 3. I don’t know **/ Sijui** | | 1  0  2 | [ ] |
| 27. | (FoI) Do you have Guidelines and operational manuals for DHFF and FFARS  **Mna miongozo ya kuendeshea *mfumo wa Usimamizi wa fedha na kutolea taarifa wa za vituo (FFARS) pamoja n***a  ***mpango wa kupeleka fedha moja kwa moja katika kituo cha kutolea huduma (DHFF)?*** | 1. No / ***Hapana*** 2. Yes / ***Ndio*** 3. I don’t know **/ Sijui** | | 1  0  2 | [ ] |
| 28. | (FoI) Do you have PFM tools to manage DHFF *(observe the availability of basic accounting and records to be maintained at heath facilities attached at the end of the checklist)*  ***Mna zana zana usimamizi wa fedha za serikali( PFM tools) kwa ajili ya mpango wa kupeleka fedha moja kwa moja katika kituo cha kutolea huduma (DHFF)? `Angalia kama zipo`*** | 1. No / ***Hapana*** 2. Yes / ***Ndio*** 3. I don’t know **/ Sijui** | | 1  0  2 | [ ] |
| 29. HCWs only. | How many times do you convene HF Management meetings and then give various response options per quarter?  Ni ***mara ngapi mnakaa vikao vya Menejimenti ya kituo kwa robo mwaka***? |  | |  | [ ] |
| 30. HCWs only. | What are the dates of the last two HF Management meetings you have conducted?  ***Taja, tarehe za vikao viwili vya mwisho vya Menejimenti ya Kituo.*** |  | |  |  |
| 31. | Do you have a HF Quality Improvement Plan (QIP)?  ***Mna mpango wa uboshaji wa huduma?*** | 1. No /***Hapana*** 2. Yes / ***Ndio*** 3. I don’t know **/ Sijui** | | 1  0  2 | [ ] |
| 32.HFGC only. | Do you endorse a Quality Improvement Plan (QIP) for your health facility?  ***Je, huwa unaidhinisha mpango wa uboreshaji wa huduma wa kituo?*** | - - 1. No /Hapana     2. I don’t know   sijui  3. Yes / Ndio | | 1  0  0.5 | [ ] |
| 33. | Do you have Annual Health Facility Plan Guideline?  ***Je, Mnamuongozo wa kuandaa mpango kazi wa kituo?*** | 1. No/***Hapana***  2. I don’t Know/ ***Sijui***  3. Yes/ ***Ndio*** | |  |  |
| 34. | Do you have annual HFP?  ***Mna mpango wa kituo wa mwaka***? | 1. No /***Hapana*** 2. Yes / ***Ndio*** | | 01  02 | [ ] |
| 35. | Budget ceilings are received on time (before November)  ***Taarifa za ukomo wa bajeti hupatikana kwa wakati*** | 1. No /***Hapana*** 2. Yes / ***Ndio*** 3. I don’t know **/ Sijui** | | 1  0  2 | [ ] |
| 36. | Head teacher from a school near by facility take part in the planning team.  ***Mwalimu Mkuu au Mwalimu mwingine hushiriki katika zoezi la uandaaji wa mpango wa Kituo.*** | 1. No /***Hapana*** 2. Yes / ***Ndio*** 3. I don’t know **/ Sijui** | | 1  0  2 | [ ] |
| 37. | Always Planning is coordinated by representative from CHMT and Technical.  ***Mchakato wa uandaaji wa mpango wa kiuo huratibiwa na Mjumbe kutoka CHM na Kamati ya Ufundi au Mratibu wa Kanda.*** | 1. No /***Hapana*** 2. Yes / ***Ndio*** | | 01  02 | [ ] |
| 38. | Member from Village/Ward Development Committee if not a member of HFGC take part in the planning team of the health facility.  ***Mjumbe kutoka kamati ya kijiji/ kata kama sio miongoni mwa wajumbe wa kamati ya usimamizi wa kituo hushiriki katika mchakato wa kuandaa mpango wa kituo.*** | 1. No /***Hapana*** 2. Yes / ***Ndio*** | | 01  02 | [ ] |
| 39. | Have you received funds for DHFF program*(if the answer is no skip questions 40, 42,43,45,46, ,47,48 and 49,)*  ***Mmeshapata fedha za mpango wa kupeleka fedha moja kwa moja katika kituo cha kutolea huduma (DHFF)?***  ***(Kama hapana neda swali la 40,42,43,45,46,47,48 na 49*)** | 1. No /***Hapana*** 2. Yes / ***Ndio*** 3. I don’t know **/ Sijui** | | 1  0  2 | [ ] |
| 40 | When did you receive money for this quarter?  ***Mlipokea lini fedha ya robo hii ya mwaka?*** | 1. Before 14^th^ of first month of the following quarter.  ***Kabla ya tarehe 14 ya mwezi wa kwanza wa robo ya mwaka iliyofuata.***  2. On 14^th^ of first month of the following quarter.  ***Manamo tarehe 14 ya mwezi wa kwanza wa robo ya mwaka iliyofuata.***  3. After 14^th^ of first month of the following quarter.  ***Baada ya tarehe 14 ya mwezi wa kwanza wa robo ya mwaka iliyofuata.***  4. Not yet received***/ Bado hatujapata.*** | | 01  02  03  04 | [ ] |
| 41 | If No Why?  ***Kama hapana kwa nini?*** | 1. We are yet to receive funding   ***Bado hatujaingiziwa fedha***   1. We are yet to be trained   ***Bado hatujapata mafunzo***   1. We are not aware at all   ***Hatuelewi*** | | 01  02  03 |  |
| 42. | What are the challenges you are facing in the course of implementing DHFF? (Request for Qualitative semi structured interview guide)  ***Je! kuna changamoto gani mnapata wakati wa utekelezaji wa mpango wa kupeleka fedha moja kwa moja katika kituo cha kutolea huduma (DHFF)*** | 1. Inadequate financial management skills.   ***Uwezo mdogo wa usimamizi wa fedha***   1. Inadequate transparent among team.   ***Kukosekana kwa uwazi***   1. Inadequate supportive supervision.   ***Usimamizi shirikishi usi wa kuridhisha***   1. Inadequate health service providers.   ***Upungufu wa watoa huduma***   1. Unsupportive relationship with HFGC. 2. Poor coordination from district level. 3. Inadequate availability of working tools. 4. Inadequate availability of accounting services. 5. Program complexity. 6. Political interfearance. 7. No challenges. 8. Other (Mention)   ***Mengineyo (taja)*** | |  | [ ] |
| 43. | In case of any challenges in the course of implementing the program, where do you report first? | 1. DMO 2. District DHFF coordinator 3. Others……(mention) | |  |  |
| 44. | What has been helpful to you in achieving DHFF goals in this Health Facility?  ***Unadhani kitu gani kimewezesha nyinyi kufanikiwa kutekeleza mpango huu wa DHFF katika kituo hiki?*** | 1. Supportive supervision   ***Usimamizi shirikishai***   1. Provision of working tools   ***Kuongeza vitendea kazi***   1. Employment of other staff   ***Kuajiri watumishi***   1. Others (mention)________   ***(Mengine taja)*** | |  |  |
| 45. | When did you receive your last supportive supervision from CHMT?  ***Ni lini ulifanyika usimamizi shirikishi wa timu ya uisimamizi wa Afya ya Wilaya (CHMT)?*** | 1. Last Quarter   ***Robo iliyopita***   1. This quarter   ***Robo hii***   1. Not yet (skip question 46)   ***Bado haujafanyika*** | | 01  02  03 | [ ] |
| 46. | During the supportive supervision did you discuss issues of DHFF implementation?  ***Katika kipindi cha ukaguzi mlijadili masuala ya DHFF?*** | 1. No 2. Yes | |  |  |
| 47. | Have you received any feedback on the previous supportive supervision?  ***Umewahi kupata mrejesho wa*** ***usimamizi shirikishi wa timu ya uisimamizi wa Afya ya Wilaya (CHMT)?*** | 1. No /***Hapana*** 2. Yes ***/ Ndio*** | | 01  02 | [ ] |
| 48. | Who is making decision of your routine Health Facility activities?  ***Nani anafanya maamuzi ya mpango wa kituo chenu?*** | 1. Matron 2. In charge 3. Any one 4. All of us through meetings. 5. Other (mention)________ | | 01  02  03  04  05 | [ ] |
| 49. | (FoI) Do you play an active role (*in charge*) / get involved *(health care workers)/* endorse *(HFGC)* (in) DHFF program implementation decision making?  ***Je unashiriki kikamilifu (mfawidhi wa kituo) / unashirikishwa kikamilifu (wahudumu wa kituo) katika maamuzi ya utekelezaji wa mpango wa kupeleka fedha moja kwa moja katika kituo cha kutolea huduma (DHFF)*** | 1. No /***Hapana*** 2. Yes / ***Ndio*** | | 01  02 | [ ] |
| 50. | What are the sources of your Health facility fund?  *(Multiple selection)*  ***Vyanzo vya fedha za kituo ni zipi?*** | 1. Health Basket Fund 2. Results Based Financing 3. Cost Sharing/ ***uchangiaji*** 4. Receipt in Kind 5. Council own sources   ***Mapato ya ndani***   1. Local Government capital Development Grant 2. Community Health Fund/TIKA 3. Implementing partner’s money 4. Other (mention)----- | | 01  02  03  04  05  06  07  08  09 | [ ] |
| 51 | Did you receive the whole amount of funds as requested in your last Financial Year Budget?  ***Kwa mwaka wa fedha uliopita mlipokea kiasi chote cha fedha mlichoomba kulingana na bajeti yenu ya kituo?*** | 1. No/***Hapana*** 2. I don’t know/ ***Sijui*** 3. Yes/ ***Ndio*** | |  |  |
| 52. | How frequently do you submit your Financial and Technical report on DHFF program implementation to the district level annualy?  ***Je, huwa mnapeleke taarifa ya utekelezaji wa mpango wa DFF wilayani kila baada ya muda gani?*** | 1. Every month   ***Kila mwezi***   1. After two months   ***Kila baada ya miezi miwili***   1. Every Three months   ***Kila baada ya miezi mitatu***   1. Other   ***Wakati mwingine taja*** | | 01.  02.  03.  04. | [ ] |
| 53. | When was your last report submission the (in question 50)? (Probe date and check where it falls along the answers provided)  ***Ni lini Mara ya mwisho kutuma taarifa zilizotajwa hapo juu? (Chunguza tarehe na onyesha inapopatikana katika majibu yaliyotolewa).*** | 1. Within seven days after the end of the quarter.  2. After seven days following end of the quarter.  3. I don’t know **/ Sijui** | | 01  02  03 | [ ] |
| 54. | Do you know Village Health Committee? *(If the answer is no skip questions 56 – 57)*  ***Unaifahamu kamati ya afya ya kijiji?*** | 1. No / ***Hapana*** 2. Yes / ***Ndio*** | | 01  02 | [ ] |
| 55. | If Yes, are you a member of that committee?  ***Kama ndio wewe ni mjumbe wa kamati hiyo?*** | 1. No / ***Hapana*** 2. Yes / ***Ndio*** | | 01  02 | [ ] |
| 56. | Have you ever attended in their meetings?  ***Umewahimkuhudhuria vikao hivyo?*** | 1. No / ***Hapana*** 2. Yes / ***Ndio*** | | 01  02 | [ ] |
| 57. | Do you work with Village Health Committee?  ***Unafanya kazi na kamati ya afya ya kijiji?*** | 1. No / ***Hapana*** 2. Yes / ***Ndio*** | | 01  02 | [ ] |
| 58. | What other programs apart form DHFF are implemented in this facility? (*List them*)  ***Ni programu gani nyingine za uboresha huduma za afya zinatekelezwa kwenye kituo hiki? (Taja)*** |  | |  |  |
|  | **Reccomendations for Improving Program Implementation** | | | |  |
| 59 | Which approach/ways/ means will you prefer to be used in submitting your reports?  ***Ni njia gani ungependelea kutumia wakati wa kutuma taarifa?*** | 1. Monthly submission as DHIS2   ***Kila mwezi kama kwenye***  ***mfumo wa DHIS2***   1. Weekly through text messages via mobile phones   ***Kwa ujumbe wa simu kila***  ***wiki***   1. On quarterly basis like any other reports for technical and financial   ***Kwa kila robo***   1. Other (mention)   ***Nyingine (taja)….*** | | 01  02  03  04 | [ ] |

**Basic accounting documents and Records to be maintained at Public Primary health facilities**

| **Documents** | **Available (Yes/No)** | **Functional /Use (Yes/No)** |
| --- | --- | --- |
| 1. Cash books (Receipt and Payments) |  |  |
| 1. Cash Receipt vouchers (ERVs) |  |  |
| 1. Issue vouchers |  |  |
| 1. Bin cards |  |  |
| 1. Payment vouchers |  |  |
| 1. Fixed Asset Registers |  |  |
| 1. Store ledger |  |  |
| 1. Cheque dispatch book |  |  |
| 1. Purchase order Books |  |  |
| 1. EFD machine for revenue collection |  |  |

**c) Health Facility In charge, Indepth Interview Guide.**

**Objectives**

- Assess to what extent the HF team is fulfilling its role, and whether this has changed with DHFF program.
- Document contextual factors that might affect intervention impact.

**Questions**

1. How long have you been working in this facility?
2. How do you know about DHFF program?
3. What is the main purpose of the DHFF program?
4. What issues to be addressed to facilitate achievemens of the DHFF program?
5. Which steps did you undertake after the introduction of DHFF program in this facility? If Yes, Probe measures undertaken (*They must include; Meeting with HFGC, HF providers meeting, Meeting with Ward/Village council)*
6. What are your roles as HF incharge in implementation of DHFF program?
7. What are the roles of the HF team as far as the DHFF program is concerned?
8. Does the facility have an annual work plan and DHFF quarterly plans? If so, how was this developed? How were you involved? What factors were considered to develop the plan? *[****Note****: use of data from HMIS for planning]*
9. What activities have you conducted as a HF team, as part of the DHFF program? (Probe: Have you received any incentives/allowances to meet?)
10. What measures do you take to ensure effective and efficient implementation of the DHFF program? (Probe issues related to participatory decision making, consultation, mentorship etc).
11. Are there any benefits from the implementation of DHFF? If Yes, Mention them.
12. Are there any other factors beyond the facility that contribute or affect DHFF implementation here? Probes: cultural issues, policies and Political issues.
13. What other programs apart from DHFF are being implemented in this health facility? How do those programs relate to DHFF?
14. What is your experience with the implementation of DHFF program? (Probe issues related to easiness of compliance).
15. In general, how do you like the way DHFF program operates? What do you like and dislike?
16. Can you tell me something about the Village Health Committee? Probe more on the composition, function and their mandate.
17. How do you work with them?

**Thanks for your time!**

**Documents to be requested:**

- HFGC meeting minutes

**d) Process evaluation Interview Guide HFGCs members**

**Objectives**

- Assess DHFF implementation progress
- Generate and investigate hypotheses on individual-level changes and changes in relationships between actors/levels (District and Facility Level) from DHFF
- Document contextual factors that might affect intervention impact

**Icebreaker**

1. How long have you been working as a chair of HFGC?
2. What are your roles as a Chair to the HFGCs? What roles do you provide?

**Theory of change**

1. What do you know about DHFF program? What is the main purpose of the DHFF program?
2. What are your roles as a chair in DHFF program implementation?
3. What issues to be addressed to facilitate achievemens of the DHFF program? (Probe more about the activities)
4. How do you communicate back to community about the implementation of DHFF in this facility?

**Context**

1. What other programs apart from DHFF program are being implemented in this facility?
2. In your experience, what have been the challenges with DHFF program implementation? How could it work better?
3. Are there any other factors beyond the facility that contribute or affect DHFF implementation here? Probes: cultural issues, policies and Political issues.
4. Do you have a Village Health Committee? If yes, how do you work with them?
5. Have HFGC team, linked to this facility received any bonus payment? If so, how many cycles? For how much?

**Thanks for your time!**

**e) In-depth interview guide for DMO and DHFF Coordinator.**

This interview should be conducted with the District DHFF Coordinator. For each interview, say this information at the start of digital recording and write this information at the top of the transcript.

First I will ask a few general questions about you, your background and your employment

| Question | Response |
| --- | --- |
| DO NOT ASK .Indicate the sex of the respondent? | Female or Male |
| What is our current job title? | ................................................................ |
| What is the highest level of education you have completed? | ................................................................. |
| Do you have any medical qualification? If yes what is it? | .......................................................... |
| How many years have you been working? | ( ) ( ) years |
| How many years have you been working in this position? | ( ) ( ) years |

**Theme 1. How DHFF will affect health care system**

I would like to ask you about how DHFF program might change health system in this district.

1. Does introduction of DHFF program has effect on your execution of your daily activities at the health facility and your role? If yes how and If no How?

Probes: new policies, awareness, effect on Planning and Budgeting, Resources availability. Are there any challenges you are facing in the course of implementing DHFF program?

1. Do you discuss issues of DHFF at the CHMT Meetings?
2. What are the role of CHSB in the implementation of DHFF?

**Theme 2. Acceptability of DHFF program**

1. What are your views about the DHFF program?
2. Please describe to me the process from start to finish of how DHFF program works (How DHFF work from the facility to the national level)
3. What are the benefits of the DHFF program?
4. How do you support HF in planning process?
5. How do health service providers respond to the available DHFF guideline?
6. How do you track the performance of the DHFF program in your Health Facilities?
7. How RHMT support you in the implementation of DHFF program? (Probe for frequency?)
8. Which steps did you undertake during introduction of DHFF program in district? Probe measures undertaken (*They must include; Meeting with HFGC, HF providers meeting, Meeting with Ward/Village council)*
9. What are the benefits associated with implementation of DHFF in your district?
10. Which approach will you prefer the HF to submit DHFF implementation reports?
11. Do budget ceilings reveled before November of every year/ If Yes/No, do you feel of any other month to be suitable for ceilings submission and why?
12. How did you handle the transaction of disbursement of funds from the previous practice to the DHFF program implementations?
13. What are your accountability options to ensure that DHFF program is implemented as per design/expectations?
14. What do you think should be done to make DHFF program successful (probe reasons)
15. Imagine that you are the Minister of Heath Community Development, Gender, Elderly and Children instilled to decide whether or not to continue with the implementation of DHFF program. What would you do?

- If says to continue, ask if they would change anything
- If says to stop, ask why

1. Can you tell me something about the Village Health Committee? Probe more on the composition, function and their mandate.
2. How do you support them?
3. Is there anyone else who you think it would it be important for me to speak to about the program?

**Thanks for your time!**

**(f)**

**Direct Health Facility Financing (DHFF): Process Monitoring, Health Facility (HF) team Focus Group Discussion (FGD) Guide**

**Objectives**

- Assess to what extent the HF team is fulfilling its role, and whether this has changed with DHFF program
- Document contextual factors that might affect intervention impact

**Questions**

1. How long have you been workers of this facility?
2. What are your roles of your HF team as far as the DHFF program is concerned?
3. In your opinion, what is the main purpose of the DHFF program?
4. How do you think the program will bring about that change or impact? Through which activities?
5. What activities have you conducted as a HF team, as part of the DHFF program? Probe: Have you received any incentives/allowances to meet?
6. Does the facility have an annual plan and DHFF quarterly plans? If so, how was this developed? How were you involved? What factors were considered? *[****Note****: use of data from HMIS for planning]*
7. How have you been involved on the use of these funds? Were you satisfied with this involvement? Why/why not? Who else was involved? (Probe: for HWs)
8. Has the implementation of DHFF affected the way staff work at this facility?

Probes: Relationships between staff; Motivation, absenteeism

1. Any changes in how the health facility operates? What do you think has contributed to these changes? (Probe: resources, incentives, financial autonomy, supervision, attention to concrete goals)
2. Any change in what health workers does or do not do? What do you think has contributed to these changes? (Probe: resources, incentives, financial autonomy, supervision, and attention to concrete goals).
3. Have there been any changes in your relationship with the CHMT or the Community? [***Note:*** *Interest in impact on referrals*]
4. Are there any challenges you are facing in the course of implementing DHFF program?
5. Have you observed any changes in the availability and management of resources at the facility level?
   1. Drug supply management
   2. Equipment
   3. Staffing
6. Are you aware of how your facility is performing in terms of maternal and Child health indicators relative to others in the district? If so, how did you become aware of this and how do you feel about this? *[****Note:*** *to check if receiving feedback from verification]*
7. What would you say about access and service utilization by the community since the start of DHFF program? (Probe: what changes have you observed? What led to these changes?)
8. What other programs apart from DHFF are being implemented in this health facility?
9. Are there any other factors beyond the facility that contribute or affect DHFF implementation here? Probes: cultural issues, policies.
10. In your experience, what have been the biggest challenges with DHFF implementation?
11. Are you aware of Village Health Committee?
12. How do you work with them?

**Thanks for your time**

**Documents to be requested:**

- HFGC meeting minutes
