# Supplementary material for: Understanding the implementation of Direct Health Facility Financing and its effect on health system performance in Tanzania: a non-controlled before and after mixed method study protocol
Source: Health Res Policy Syst. 2019 Jan 30;17:11. doi: 10.1186/s12961-018-0400-3 (PMC6354343; doi:10.1186/s12961-018-0400-3)
Supplement: Supplementary file 2 — Tools for assessing the effects of the DHFF programme (English/Kiswahili). (DOC 376 kb) [file 12961_2018_400_MOESM2_ESM.doc]

Appendix No. 1(a) Questionnaire to be administered to health facility In-charge

***Kiambatanisho namba 1(a) Dodoso la msimamizi wa kituo cha huduma ya afya***

Objective No. 1 (Impact evaluation): Structural Quantity maternal health services indicators.

***Lengo Namba 1 (tathmini ya matokeo): Viashiria vya muundo wa ubora wa huduma za afya ya uzazi.***

QUANTITY AND QUALITY HEALTH CHECKLIST (Selected health system performance indicators); Adopted from RBF program.

***DODOSO LA KUHAKIKI UBORA WA HUDUMA ZA AFYA (viashiria vya utendaji wa mfumo wa afya); vimechaguliwa kutoka “Program ya Malipo kwa Matokeo”***

| Health Facility Name:  ***Jina la Kituo:*** | | Assessed Quarter: ……… Year: ……….  ***Robo iliyotathminiwa: Mwaka ….*** |
| --- | --- | --- |
| District:  ***Wilaya :*** | | Region:  ***Mkoa:*** |
| Phone No:  ***Namba ya simu:*** | Fax:  ***Nukushi:*** | P.O Box:  ***S.L.P*** |
| Status: Public: Missionary : Private : Partner:  ***Hali : Serikali***  ***Misheni:*** ***Binafsi***  ***Mbia:*** | | |
| Catchment Population:  ***Idadi ya walengwa :*** | | Number of beds:  ***Idadi ya vitanda:*** |
| Name of In-charge:  ***Jina la Mfawidhi:*** | | Phone No:  ***Namba ya simu:*** |
| P.O. Box:  ***S.L.P:*** | E-mail:  ***Barua pepe:*** | |

| **NO** | **Service category** | **Indicator** | **Type of Indicator** | **Description/ definition of indicator** | **Source of data** |
| --- | --- | --- | --- | --- | --- |
|  | Staffing | Number of staff in this facility | Service availability | Number of staffs in that facility in a given period of time | Health Facility profile. |
|  | Staffing cadre | Number of staff in this facility per cadre | Service availability | Number of staffs in that facility per cadre in a given period of time | Health facility profile |
|  | Outpatient | Number of new outpatient consultations | Service Utilization | Number of new cases or patients (New diagnosis) attending and receiving outpatient services during the quarter | HMIS – OPD Register Book 5 |
|  | RCHS | Number of first antenatal visits, with gestation age <12 weeks. | Service Utilization | Number of women starting ANC before 12 weeks of gestation age at the health facility. | HMIS – ANC Register book 6 |
|  | RCHS | Number of pregnant women attending ANC at least 4 times during pregnancy. | Service Utilization | The number of pregnant women receiving fourth ANC consultation with a health professional at the facility or through outreach by facility staff at the particular quarter. | HMIS – Register book 6 |
|  | Reproductive and Child Health | Number of pregnant women receiving 2+ doses of treatment presumptive treatment of malaria | Service availability | Number of pregnant women receiving 2+ doses of SP during pregnancy at that particular quarter. | HMIS – ANC Register book 6 |
|  | Reproductive and Child Health | Number of HIV infected pregnant women receiving ARVs for PMTCT | Service availability | Number of HIV infected pregnant women receiving ARVs Prophylaxis for PMTCT at health in that particular quarter | HMIS – ANC Register book 6/ ART Register |
|  | Reproductive and Child health | Number of institutional deliveries | Service Utilization | Number of deliveries conducted at the health facility and attended by a health professional (MD, Midwife, RN, EN, CO, CA) during the period of that quarter. | HMIS Book 12 |
|  | Reproductive and Child health | Number of new users on modern Family Planning methods | Service availability | Number of newly accepting contraception by pills, injection, implant, IUCD, at the facility or through outreach and CBD within the particular quarter. | HMIS- Family Planning Register book 8 |
|  | Reproductive and Child health | Number of pregnant mothers receiving Mebendazole for de-worming | Service availability | Number of pregnant women given mebendazole in the evaluated quarter at the health facility during pregnancy (20 weeks and above) | ANC Register Book 6 |
|  | Reproductive and Child health | Number of postnatal mothers receiving Post Natal Care services within 3-7 days after delivery | Service utilization | Number of women receiving post natal care at the facility within seven day after delivery | HMIS – Post Natal Register Book 13 |
|  | HIV/AIDS | Number of clients initiated by heath care provider to counsel and Test for HIV (PITC) | Service utilization | Number of patients/clients attended at the health facility and initiated by the health provider to test for HIV | ART Register |

**Appendix No. 1 (b); Checklist for observation in health facility.**

***Kiambatanisho nambari 1(b); Nyezo ya tathmini ya kituo cha huduma ya afya.***

Objective No. 1 (Impact evaluation): Structural quality maternal health services indicators

***Lengo Namba 1 (tathmini ya matokeo): Viashiria vya muundo wa ubora wa huduma za afya ya uzazi.***

| Health Facility Name:  ***Jina la Kituo:*** | | | Assessed Quarter: ……… Year: ……….  ***Robo iliyotathminiwa: Mwaka ….*** |
| --- | --- | --- | --- |
| District: ***Wilaya :*** | | | Region: ***Mkoa:*** |
| Catchment Population:  ***Idadi ya walengwa :*** | | | Number of beds:  ***Idadi ya vitanda:*** |
| How long have you been working in this facility?  ***Umefanya kazi kwa muda gani kwenye hiki kituo?*** | | | 1. ***1 – 3 years*** 2. ***4 – 6 years*** |
| Phone No of incharge:  ***Namba ya simu ya mganga mfawidgi wa kituo:*** | | | |
| Highest level of education  ***Kiwango cha juu cha elimu*** | 1.Primary  ***Msingi***  2.Secondary  ***Sekondari***  3. Certificate  ***Astashahada***  4.Diploma  ***Stashahada***  5.Advanced diploma  ***Stashahada ya juu***  6.University degree  ***Shahada***  7.Masters  ***Shahada ya uzamili***  8. Others  ***Nyingine*** |  | |

***Lengo Namba 1 (tathmini ya matokeo): Viashiria vya muundo wa ubora wa huduma za afya ya uzazi.***

| SN  ***NA*** | DIMENSION  ***ENEO*** | INDICATOR CHECKLIST ELEMENTS &MEANS OF VERIFICATION  ***VIGEZO*** | CRITERIA  ***JINSI YA KUHAKIKI*** | POSSIBLE MAX SCORE  ***ALAMA ZA JUU*** | OBTAINED SCORE  ***ALAMA ZILIZOPATIKANA*** |
| --- | --- | --- | --- | --- | --- |
| 1 | PRIVACY  ***FARAGHA*** | Privacy in Individual treatment /service delivery rooms have full privacy during service provision  ***Faragha wakati wa kumtibu mgonjwa/ vyumba vya kutolea huduma vina faragha ya kutosha***   - All service delivery rooms with doors that close   ***Vyumba vyote vya kutoa huduma vina milango inayofunga/yenye vitasa***   - All service delivery rooms with screen to partition the examination area/bed   ***Vyumba vyoote vya kutolea huduma vina pazia la kuzuia eneo la kutolea huduma/kitanda***   - Windows with curtains or painted or with frosted glass   ***Madirisha yenye mapazia au yenye rangi au kioo usichoweza ona ndani***   - Divider (screen/curtain) if the service delivery room is shared.   ***Kitenganishi (kioo/pazia) kama chumba cha huduma kinatumiwa na mtu zaidi ya mmoja.*** | Privacy Assured in rooms and all criteria met = 4  ***Faragha niya uhakika katika vyumba vyote na vigezo vyote vimezingatiwa =4***  Service room with all criteria = Number of rooms fulfilling all criteria X 4/Number of available rooms  Privacy not assured=0  ***Vyumba vya huduma vyenye vigezo/sifa zote = Idadi ya vyumba vinavyo kidhi vigezo vyote X 4/vyumba vilivyopo.***  ***Faragha haijasibitishwa =0*** | **4** |  |
| 2 | HYGIENE AND SANITATION  ***AFYA NA USAFI WA MAZINGIRA*** | 2a)*Presence of clean and functioning disinfected toilet/s for patients, staffs and physically challenged people:*  ***Upatikanaji wa vyoo safi na salama kwa wagonjwa, watumishi na walemavu wa viungo;***  **1)** Functioning toilet [VIP latrine which is not full OR a flushing toilet with working or improvised flushing system],  ***Choo kinachofanya kazi (choo bora cha shimo ambacho hakijajaa au choo cha maji ambacho mfumo wa kusafisha choo unafanya kazi)***  **2)** Toilets clean inside and out with no stagnant water and no foul smells],  ***Choo safi nje na ndani ambacho hakina maji yaliyotwama wala harufu mbaya.***  **3)** Presence of toilet paper or water. And in case of flushing toilet, a dust bin.  ***Upatikanaji wa karatasi laini au maji. Na kama choo cha mfumo wa maji basi kuwa na chombo cha taka.***  **4)** Hand-washing facilities just outside the toilet or with basin inside toilet [soap and source of water]  ***Chombo cha kunawia mikono nje ya choo au sinki la ndani (sabuni na maji)*** | All element available for all toilets = 4  ***Vipengele vyote katika vyo vinapatikana = 4***  Element missing for one or all toilets = Deduct 1 Point per missing element  ***Kipengele kimoja kikosekana kwa vyo vyote au kimoja = toa alama 1 kwa kila kipengele kilichokosekana*** | **7** |  |
|  |  | **2b.** No organic waste, used syringes, needles, used bandages or dangerous products on the ground of the facility that are easily accessible to the public (including waste pit area) and grounds surrounding the HF entirely cleared of weeds and stagnant water drained  ***Kusiwe na taka ngumu, mabomba ya sindanoyaliyotumika au vitu vya hatari kwenye kumbi, vyumba au mahali pengine kokote kwenye maeneo ambayo hufikiwa na watu(ikiwemo sehemu ya kutupa taka) na eneo la kuzunguka kituo linatakiwa liwe safi na halina maji yaliyo tuwama.*** | Yes = 1  ***Ndio = 1***  If present = 0/1  ***Kama vipo =0/1*** |  |  |
|  |  | **2c.** Presence of: Functioning incinerator, fenced in and ash pit.  If no incinerator, it must have waste pit with evidence of use by burn and bury but also fenced in.  Presence of placenta pit with slab and cover ***Kuwapo kwa tanuru la kuchomea taka*** ***linalofanya kazi na lililozungushiwa uzio na lina sehemu ya kutupa majivu. Endapo kituo hakina tanuru la kuchomea taka ni lazima kituo kiwe na shimo la kuchomea taka na kuzifukia lililozungushiwa uzio.***  ***Kuwepo na shimo la kutupa kondo la nyuma lenye mfuniko.*** | Functioning incinerator=2  ***Tanuru linalofanya kazi au shimo la taka lililozungushiwa uzio = 1***  If it is Placenta pit in use meeting all criteria = 1  ***Kama kunashimo la kutupa kondo na lina vipengele vyote = 1***  If not available =0  ***Kama hakuna = 0*** |  |  |
| **3** | LABOUR WARD  ***WODI YA KUJIFUNGULIA*** | Delivery room with essential equipment and supplies for quality service delivery:  ***Chumba cha kujifungufungulia chenye vifaa tiba vyote muhimu kwa utoaji wa huduma bora ya afya.***  **A.** Delivery bed functional, Clean, Adjustable, with a footstool,  ***Kitanda cha kujifungulia kinafanya kazi, kisafi, kurekebishika na chenye ngazi ya kupandia na kisicho na kutu.*** | All delivery bed meeting all criteria = 1  ***Vitanda vyote vya kujifungulia vimekidhi vigezo vyote = 1***  If not all=1/#beds*bed meeting criteria  ***Kama sio vyote = 1/idadi ya vitanda*vitanda vyenye vigezo vyote***  If none meets criteria=0  ***Kama hakuna kinacho kidhi vigezo =0*** | **14** |  |
|  |  | **B.1)** One Functional gouse neck lamp / light source, **2)** one Functional newborn weighing scale, **3)** One Drum with Sterile gauze (with date and closed) and, **4)** One drum with cotton wool (with date and closed), **5)** One Suction machine, **6)** Resuscitation kit (Ambu-bag, tubes), **7)** One Mackintosh (plastic mattress cover or disposable) for each delivery bed  ***1)Taa 1 maalumu / tochi 1 inayofanya kazi (chanzo cha mwanga)***  ***2)Mzani 1 wa kumpima motto mchanga unaofanya kazi 3) Dramu moja lenye vifaa tasikama vile shashi 4)Dramu 1 lenye pamba***  ***5)Mashine moja ya kutolea mchojozo kwa mototo 6)Kifaa cha kumpa hewa motto 7)Mpira 1 wa kufunika kitanda kwenye kitanda cha kujifungulia.*** | All elements Available element = 7  Missing element = Deduct 1 point per missing element  ***Uwepo wa vipengele vyote =7***  ***Kukosekana kwa kipengele kimoja toa maksi 1*** |  |  |
|  |  | **B)** At least 2 sterilized delivery trays (1 kidney dish or gallipot, 2 scissors, 2 clamp forceps, 1 stitching forceps, 1 dissecting forceps, 1 sponge holding forceps per tray, 2 needles with suture, 1 umbilical cord clamp)  ***Angalau trei 2 za kuzalishia ambazo zimetasiswa kila trei ndani liwe na (kibeseni kimoja chenye umbo la figo/kikombe, mikasi 2, foseps 2 za kubania kitovu, foseps moja ya kushikilia nyuzi, foseps moja ya kushikilia shashi/pamba na sindano 2 za kushonea na nyuzi zake, na kamba moja ya kufunga kitovu)*** | All elements Available element =3  If one is not available = 0 ***Vigezo vyote vimetimia = 3***  ***Kama hata kimoja hakuna =0/3*** |  |  |
|  |  | **C)** PPEs:  **Two** Plastic aprons, **Two** pairs Gumboots/closed shoes, **Two** Masks, **Two** Goggles, At least **one** full or nearly full box of Clean (50 pairs), **one** full or nearly full Sterile gloves (50 pairs)  ***Vifaa vya kinga Binafsi***  ***Aproni za plastiki 2, Jozi 2 za buti,/Viatu vilyofungwa, Barakoa 2, Kingajicho 2, Angalau boksi 1 za glovu safi zilizojaa au karibu ya kujaa (Jozi 50), Glovu tasi boksi zilizojaa (Jozi 50)*** | All element available=3  Even one missing= 0/3  ***Vipengele vyote vipo = 3***  ***Kikikosekana kipengele kimoja = 0/3*** |  |  |
| 4 | OBSTETRIC EMERGENCIES  ***(Swali la Kituo cha Afya) HUDUMA YA DHARURA WAKATI NA BAADA YA KUJIFUNGUA*** | **Availability of appropriate equipment and materials available to treat/manage patients with obstetric emergencies:**   1. Sterilized manual removal aspiration kit available [MVA kits2] ***(Health centre only)***   ***Upatikanaji wa vifaa tiba sahihi vya kutolea huduma ya dharura wakati na baada ya kujifungua***  ***Upatikanji wa vitendanishi vilivyo salama kwaajili ya huduma [Kitendanishi cha MVA 2]*** | MVA kits available = 1  ***Not available = 0***  ***Kitendanishi cha MVA kipo = 1***  ***Hakipo = 0*** | **30** |  |
|  |  | 1. Blood transfusion facilities available [Blood bank with 5 units different groups preferably O-group of blood available] ***(health centre only)***   ***Upatikanaji wa huduma ya kuongezewa damu [benki ya damu yenye uniti 5 za aina tofauti za makundi ya damu ikiwemo aina ya kundi O]*** | All O-groups available=5  O groups missing=0/5  All other groups available=5  All other groups missing=0/5  ***Uwepo wa uniti zote 5 kuwa aina ya kundi O/ makundi yote (A.B,AB, & O) = 5***  ***Kuwepo kwa makundi yote ya damu bila kundi “O” = 0*** |  |  |
|  |  | **C) Presence of:-**  **1)** Vacuum extractor, ***(Health centre only)***  **2)** Gun let gloves for manual removal of placenta  ***1)Uwepo wa chombo cha kuvutia motto, 2)Glovu ndefu za kutolea kondo la nyuma*** | 2 elements available = 4  One missing = deduct 2  ***Uwepo wa vipengele vyote 2= 4***  ***Kipengele kimoja kikikosekana = Punguza 2.*** |  |  |
|  |  | **D) 1)** Suction machine, **2)** Resuscitation kit [Ambu bags different sizes, Sodium bicarbonate, Vit. K]  ***1)Chombo cha kumfyonza michojozo kutoka kwa motto 2) Vifaa vya kufufulia mtoto / mama viwepo kama vile Ambubags za saizi mbali mbali, Sodium bicarbonate, Vit.K*** | 2 elements available= 6  One missing/incomplete= Deduct 3  ***Vipengele vyote viwili vipo = 6***  ***Kikikosekana kipengele kimoja punguza = 3*** |  |  |
|  |  | **E) Infusions:**  ***1)*** Three bottles of Ringer lactate, Three bottles of Normal saline 1000mls, ***2)*** Sets of cannula, Gauge 14 & 18  ***Kuwepo kwa maji ya dripu;***  ***A)Chupa 3 za Ringer lactate, Chupa 3 za Normal saline 1000mls,***  ***B)Seti ya Kanula ya saizi 14 & 18*** | A&B available = 4  A/B missing = Deduct 2  ***A&B vikiwepo = 4***  ***A/B ikikosekana = punguza 2.*** |  |  |
|  |  | **Infusions (cont.):**  ***3)*** Giving sets 3, ***4)*** Syringes 2cc, 5cc, 10cc [5,5,5]  ***Dripu (endelea)***  ***C)Seti 3 za kuweka dripu, D) Mabomba ya sindano ya 2cc, 5cc, 10 cc [5,5,5]*** | C & D available = 2  C/D missing / incomplete = Deduct 1  ***C&D vikiwepo = 4***  ***C/D ikikosekana = Punguza 2*** |  |  |
|  |  | ***F) Emergency Medicines Availability:***  1) Magnesium Sulphate, 2)Nifedipine/Hydralazine  ***Uwepo wa dawa za dharura***  ***1) Magnesium Sulphate, 2)Nifedipine/Hydralazine*** | Both available = 4  One missing = Deduct 2  ***Zote zipo =4***  ***Moja hakuna = punguza 2*** |  |  |
|  |  | **G) Antibiotics:**  **1)** Metronidazole Inj., **2)** Ampicillin inj. OR Gentamycin inj. **3)** Ceftriaxone inj. 4) Oxytocin  ***Viua vijisumu: (rejea sampuli 4 za dawa tajwa juu)*** | These four drugs available = 4  Missing drug= Deduct 1  ***Dawa 4 zikiwepo = 4***  ***Ikikosekana dawa = punguza 1*** |  |  |
|  |  | **H) Sedatives:**  (E.g. diazepam)  ***“Sedatives” Mfano : Diazepam*** | Sedative Available = 1  Not available = 0/1  ***Ikiwepo = 1***  ***Hakuna = 0/1*** |  |  |
| 5 | WASTE MANAGEMENT  ***UTUPAJI SAHIHI WA TAKA*** | **Waste management done as per standard guidelines in clinical procedures rooms:**  Availability of:  ***4.1)Labor ward and dressing room:***  Three buckets, each bucket clearly labeled with today’s date, 1 bucket with chlorine 0.5%, 1 with soapy water and 1 with clean water;  ***Utupaji taka unafanyika kwa kuzingatia miongozo ya vyumba vya tiba:***  ***Uwepo wa:***  ***Kwenye wodi ya kujifungulia na chumba cha upasuaji: Ndoo tatu kila ndoo iwekwe alama ya tarehe ya leo, ndoo moja klorini 0.5%, 1 maji ya sabuni na 1 maji safi.*** | 3 buckets available = 1  Not available or not meeting criteria = 0/1  ***Ndoo 3 zipo = 1***  ***Hazipo au hazijakidhi vigezo =0/1*** | **6** |  |
|  |  | **2)*Inpatient wards (Including labor ward, laboratory and immunization/Injection room):***  At least 1 safety box with sharps not exceeding ¾ full, and no sharps sitting on top of the box  ***Wodi ya kulaza wagonjwa (ikiwemo wodi ya wazazi, maabara na sehemu ya kutolea Chanjo/ sindano): Angalau boksi salama moja la vifaa vyenye ncha kali ambalo linatakiwa lisijae kufikia ¾, kusiwe na taka zinazoonekana nje ya kasha.*** | Available and criteria met=2  If partial= 0/2  ***Uwepo wa vigezo vyote = 2***  ***Kama havijakamilika = 0/2*** |  |  |
|  |  | 3) *In labor ward, laboratory and minor theatre:*  Proper waste segregation using Red, Yellow and Black/Blue bins with color coded bin liners – labeled bin liners ok in lieu of colored  ***Kwenye wodi ya kujifungulia, maabara ma chumba cha upasuaji***  ***Utenganishaji wa taka ufanyike kwa kutumia vifaa vyenye rangi nyekundu, njano na nyeusi/ bluu navyo viwe na mifuko laini yenye rangi nyekundu, njano na bluu kulingana na vifaa vyake.*** | Waste segregation meeting criteria and in all relevant rooms= 3  If partial= 0/3  ***Utenganishaji wa Taka unafuata vigezo katika vyumba vyote = 3***  ***Kama haijakamilika = 0/3*** |  |  |
| **6** | STERILIZATION  ***UTASISHAJI WA VIFAA*** | **Availability of proper sterilization of instruments:**  **1.** Existence of proper means / methods of sterilizing instruments:  ***Steam sterilization (Autoclave) or Dry heat sterilization or Chemical sterilization***  **2.** SOPs for sterilization displayed on the wall by the equipment  **3.** Each pack has an indicator for control of sterility *(litmus or date of sterilization)*  ***Upatikanaji wa mbinu/njia zinazofaa za kutasisha vifaa:***   - ***Kutasisha kwa kutumia mvuke ( Autoclave) au Joto kali au kemikali*** - ***Hatua za utasishaji zilizobandikwa ukutani kwa vifaa maalumu*** - ***Kila kifurushi iwe na kifaa kinachoonyesha namna ya kutambua kuwa vifaa ni tasi (tarehe)*** | Each element fulfilling the criteria = 1  Criteria not met = 0/1  ***Kila kipengele kinachokidhi vigezo=1***  ***Vigezo havijafikiwa=0/1*** | **3** |  |
| **7** | MATERNAL DEATH AUDITS  ***UHAKIKI WA VIFO VYA UZAZI*** | **Proportion of maternal deaths in HFs that are completely and appropriately audited and action plan in place:**  A) Select one audited case and check if they were; 1) Completely, 2) Correctly filled and 3) Action plan in place.  ***B) Chagua bila mpangilio vifo 3 vilivyotokea na angalia kama 1)Fomu zimejazwa kote 2)Fomu zimejazwa kwa usahihi 3) Mpango mkakati upo.*** | A case with 3 criteria = 10  Even one criteria missing = 0/10  ***Kifo kinachokidhi vipengele vyote 3 = 10***  ***Kifo kisichokidhi hata kipengele 1 = 0/10*** |  |  |
|  |  | B) In cases where the facility has no maternal deaths, staff should hold meetings and discuss strategies should be in place to ensure that the community do not experience deaths in the next quarter  Assess if the strategies are in place with emphasis on   - ANC clinic - Labour and delivery - Post-natal care - Patient/Community factors   ***C) Katika vituo ambavyo hakuna vifo, watumishi wa vituo waitishe mikutano na wananchi kujadili mikakati ya kuondoa vifo vitokanavyo na uzazi ili jamii isipate vifo katika robo inayofuata.***  ***Hakiki kama mikakati iliyopo ina weka msisitizo kwenye***   - ***Kliniki ya mama na mtoto*** - ***Huduma ya kujifungua*** - ***Huduma baada ya kujifungua*** - ***Mgonjwa/ sababu za kijamii*** | If meeting minutes and well-structured with 4 strategies available = 15  Even if one Strategy not discussed = 0 /15  ***Uwepo wa muhtasari wa kikao unaogusa mikakati yote 4 = 15***  ***Kama mkakati hata mmoja haujajadiliwa =0/15*** |  |  |

**Appendix No. 2(a) Questionnaire to HFGC Chairperson**

***Kiambatanisho Na.2 (a) Dodoso la mwenyekiti wa kamati ya usimamizi wa kituo.***

**Objective No. 2 (Impact evaluation):** To assess the effect of DHFF program implementation on the accountability and governance of Health Facility Governing Committee (HFGC).

***Lengo Na.2 (Uhakiki wa matokeo): Kutathmini matokeo ya utekelezaji wa mfumo wa kupeleka fedha moja kwa moja vituo vya afya kwenye usimamizi wa uwajibikaji na utawala bora wa kamati za usimamizi wa vituo vya afya.***

Serial No. **[__|__|__|__]**

***Nambari ya kumbukumbu.***

Name of the Region: _____________________________________

***Jina la Mkoa:***

Name of the District Council: ______________________________

***Jina la Wilaya***

Name of the Health Facility: _______________________________

***Jina la Kituo***

Type of Health Facility: **01**= Dispensary **[ ] 02=** Health center **[ ]**

***Aina ya Kituo 01 = Zahanati [ ] 02 = Kituo cha Afya [ ]***

Please put the appropriate number of a response in the given *box.*

***Tafadhali weka namba ya jibu sahihi kwenye chumba kilichotolewa***

| **SN**  ***Na.*** | | **Questions**  ***Maswali*** | **Responses**  ***Majibu*** | **Code** | **Code** |
| --- | --- | --- | --- | --- | --- |
| **SECTION A: DEMOGRAPHIC INFORMATION**  ***KIPENGELE A: TAARIFA ZA AWALI ZA MDODOSWAJI.*** | | | | | |
| 1. | (a)Sex  ***Jinsi*** | | 1.Male  ***Mwanaume***    2.Female  ***Mwanamke*** | 1  2 | [ ] |
|  | (b)How old are you?  ***Una umri gani?*** | | [________] |  |  |
|  | Marital status  ***Hali ya ndoa*** | | 1.Married  ***Nipo kwenye ndoa***  2.Cohabiting  ***Tunaishi bila ndoa***  3.Single  ***Sina ndoa***  4.Divorced/Separated  ***Mtalaka***  5.Widow/widowed  ***Mjane / Mgane*** | 1  2  3  4  5 | [ ] |
|  | Highest level of education  Kiwango cha juu cha elimu | | 1.Primary  ***Msingi***  2.Secondary  ***Sekondari***  3. Certificate  ***Astashahada***  4.Diploma  ***Stashahada***  5.Advanced diploma  ***Stashahada ya juu***  6.University degree  ***Shahada***  7.Masters  ***Shahada ya uzamili***  8. Others  ***Nyingine*** | 1  2  3  4  5  6  7  8 | [ ] |
|  | Do you convene meetings as per guideline (quarterly meetings)?  ***Je! Vikao huitishwa kwa mujibu wa miongozo (vikao vya robo mwaka)?*** | | 1.Yes  2. No | 1  0 |  |
|  | Do you present agenda prior to the meeting?  ***Je! huwa mnapata ajenda kabla ya kikao?*** | | 1. Yes 2. No | 1  0 |  |
|  | Do you take part in HF Planning and Budgeting exercise?  ***Je! huwa unashiriki zoezi la kuandaa mpango na bajeti ya kituo?*** | | 1. Yes 2. No | 1  0 |  |
|  | Were you trained on Planning and Budgeting?  ***Je ulipatiwa mafunzo ya kuandaa mpango na bajeti ya kituo?*** | | 1. Yes 2. No | 1  0 |  |
|  | Do you participate in Plan prioritization meeting with Health Facility Management Team (HFMT)?  ***Je huwa unashiriki katika mchakato wa kuandaa vipaumbele kwa kushirikiana na menejimenti ya kituo?*** | | 1. Yes 2. No | 1  0 |  |
|  | Does Management of the facility share feedback from CHMTs with HFGC to know the amount of fund allocated for the implementation of plans?  ***Je menejimenti ya kituo hutoa mrejesho toka Timu ya Afya ya Wilaya kwa Kamati ya usimamizi wa kituo juu ya fedha zilizo pitishwa kwaajili ya kutekeleza mpango wa kituo?*** | | 1. Yes   2. No | 1  0 |  |
|  | Does HFGC receive, review and approve financial report of the facility in quarterly basis?  ***Je kamati ya usimamizi wa kituo hupokea, kupitia na kuridhia taarifa za fedha za kituo za kila robo mwaka?*** | | 1. Yes 2. No | 1  0 |  |
|  | Does Data collected and reported used for improving the health provision, decision making and planning purposes?  ***Je taarifa zinazo kusanywa na kuripotiwa hutumika kuleta maboresho ya utoaji wa Huduma, maamuzi na mipango ya kituo?*** | | 1. Yes 2. No | 1  0 |  |
|  | Are you aware of the DHFF programme?  If the answer is No, skip question 13 to 15.  ***Je, unafahamu kuhusu utaratibu wa kupeleka fedha moja kwa moja kwenye vituo vya afya?***  ***Kama jibu ni HAPANA, ruka swali namba 13 mpaka 15.*** | | 1. Yes 2. No | 1  0 |  |
|  | Do you think Transition/decentralization of facility fund disbursement from District consolidated account to direct health facility-financing (direct disbursement to facility account) will/ has increase assurance of sufficient revenues to the facility level?  ***Je unafikiri maboresho/ mabadiliko toka mfumo wa ugatuzi wa kupeleka fedha kwenye vituo toka mfumo wa kupeleka fedha kwenye akaunti za wilaya utaongeza uhakika wa upatikanaji wa fedha katika vituo vya afya?*** | | 1. Yes   2. No | 1  0 |  |
|  | Do you think DHFF (will) increase accountability on resource use?  ***Je unafikiri mfumo wa kupeleka fedha moja kwa moja kwenye vituo utaongeza uwajibikaji kwenye usimamizi wa fedha?*** | | 1. Yes 2. No | 1  0 |  |
|  | Do you think DHFF (will) reduce overlap in the use of fund at facility level?  ***Je unadhani mfumo wa kupeleka fedha moja kwa moja kwenye vituo utapunguza mwingiliano wa matumizi ya fedha katika vituo?*** | | 1. Yes 2. No | 1  0 |  |

**Checklist for Objective No.2**

***Hojaji la lengo Na.2 ; Hojaji kwa ajili ya mwenyekiti wa kamati ya usimamizi wa kituo.***

| S/No  ***Na.*** | DIMENSION  ***ENEO*** | INDICATOR CHECK LIST, ELEMNTS AND MEANS OF VERIFICATION  ***VIGEZO*** | CRITERIA  ***JINSI YA KUHAKIKI*** | POSSIBLE MAX SCORE  ***ALAMA ZA JUU*** | OBTAINED SCORE  ***ALAMA ZILIZOPATIKANA*** |
| --- | --- | --- | --- | --- | --- |
| 1 | SOCIAL ACCOUNTABILITY:  ***UWAJIBIKAJI*** | Transparence in operations and information sharing:  Presence of the following displayed at the notice board;  1) Price list for services displayed (Inc. free services; 2) Quarterly Income and Expenditures reports displayed; 3) Health Facility Governing Committee meetings conducted and minutes available and filed; 4) Working hours displayed for outpatient services; 5) Mobile/phone number and names for complaints displayed  ***Kituo kinazingatia uwazi katika kutekeleza shughuli zake na kushirikisha taarifa zake:***  ***Angalia uwepo wa vitu vifuatavyo vilivyobandikwa kwenye mbao za matangazo;***  ***1)Bei za huduma zimebandikwa (Ni pamoja na huduma zitolewazo bure)***  ***2)Taarifa ya mapato na matumizi zimebandikwa kila robo mwaka.***  ***3)Mikutano ya Kamati ya usimamizi wa kituo iliyofanyika na kumbukumbu za kikao zipo kwenye jalada maalumu.***  ***4)Saa za kazi kwa ajili ya kuwahudumia wagonjwa zimebandikwa.***  ***5)Namba za simu za mkononi na mezani na majina kwa ajili ya kuwasilisha malalamiko zimebandikwa.*** | Presence of all 5 elements = 5  Even one missing element = 0/5  ***Vipengele vyote 5 vikiwepo=5***  ***Kikikosekana kipengele hata kimoja=0/5*** | **5** |  |
| 2. | HFGC  ***Kamati ya usimamizi wa kituo cha huduma ya afya*** | 1) Availability of HFGC register  ***Uwepo wa kitabu cha mahudhurio cha wajumbe wa kamati ya usimamizi wa kituo.*** | Presence of all 4 elements = 4  Even one missing element = 0/4  ***Vipengele vyote 4 vikiwepo=4***  ***Kikikosekana kipengele hata kimoja=0/4*** | **4** |  |
|  |  | 2)Number of times you convened meetings last year  ***Idadi ya vikao vilivyofanyika mwaka jana*** |  |  |  |
|  |  | 3)Existence of gender representation in the HFGC team (at least 3 members should be women).  ***Uwepo wa uwakilishi wa kijinsia katika kamati (angalau wajumbe 3 wawe wanawake).*** |  |  |  |
|  |  | 4)Availability of operational guideline.  ***Uwepo wa miongozo ya uundwaji na uendeshaji wa kamati za vituo.*** |  |  |  |
|  |  | **Available minutes of the Health Facility Governing Committee (HFGC) for the quarter**; Minutes should contain:  1) Date and time indicated 2)Agenda available 3)Meeting minutes available for assessed quarter, 4) Attendance list and signatures available, 5) Evidence of use of DHFF funds discussed during the meeting, 6) Evidence of discussion of challenges confronting the facility and action points documented  ***Uwepo wa kumbukumbu za vikao vya kamati ya usimamizi wa kituo za robo iliyofanyiwa tathmini;***  ***Kumbukumbu za vikao zijumuishe;***  ***1)Tarehe na muda wa kikao 2) Ajenda za kikao 3) Kumbukumbu za vikao kwa robo mwaka iliyofanyiwa tathmini 4) orodha ya mahudhurio na saini zilizopo 5)ushahidi wa matumizi ya fedha za kituo 6) ushahidi wa majadiliano ya changamoto zinazokikumba kituo na hatua za kutatua.*** | Each element available = 1  ***Kila uwepo wa kipengele kimoja =1***  Missing element=Deduct 1  ***Kipengele kinachokosekana = punguza maksi 1***  Note: This applies if 1&2 are available.  ***Kumbuka: Hii inahusika kama 1&2 zipo.*** | **6** |  |
|  |  | **Availability of Facility Progress Report:**  Verify previous Quarter Facility Progress Report, including:  **1)** Technical Report (Including Annual work Plan Implementation), **2)** Financial Report  ***Uwepo wa taarifa za utendaji wa kituo;***  ***Hakiki utendaji wa kituo kwa robo mwaka iliyopita:***  ***1)Taarifa za Kitaalamu (ikiwemo utekelezaji wa mpango wa mwaka wa kituo)***  ***2)Taarifa za fedha*** | Both elements available in the report = 4  ***Endapo vipengele vyote vipo = 4***  Even one missing = 0/4  ***Endapo hata kipengele kimoja kitakosekana = 0/4*** | **4** |  |
|  |  | Medicine and Equipment consignment receipt  ***Rejista ya upokeaji dawa na vifaa tiba***  Witnessed its receipt 1  ***Risiti ilikubaliwa 1***  Did not witness its receipt 2  ***Risiti haikukubaliwa 2***  Verify the presence of signature in the Visitors books  ***Hakiki uwepo wa saini kwenye daftari/kitabu cha wageni.*** | ***Rejista ipo na risiti imetolewa – 1***  ***Kitabu cha wageni kimesainiwa – 1***  ***Kukesekana kwa kipengele kimoja = 0/2*** | **2** |  |

**Appendix 2 (b) Interview guide for Chairman of HFGCs**

***Mwongozo wa hojaji la wenyekiti wa kamati ya usimamizi wa kituo cha afya.***

**Direct Health Facility Financing (DHFF) program implementation in Tanzania: Process evaluation Interview Guide**

***Utekelezaji wa program ya upelekaji wa fedha za huduma ya afya moja kwa moja kwenye vituo vya afya nchini Tanzania: Hojaji la tathmini ya mchakato.***

**Objectives**

**Malengo**

- Assess DHFF implementation progress
- Generate and investigate hypotheses on individual-level changes and changes in relationships between actors/levels (District and Facility Level)from DHFF
- Document contextual factors that might affect intervention impact

**Icebreaker**

1. How long have you been working as a chair of HFGC in this facility?

***Ni kwa muda gani umefanya kazi kama Mwenyekiti wa wa kamati ya usimamizi wa kituo***

1. What are your roles as a Chair to the HFGCs? What roles do you provide?

***Kama Mwenyekiti wa kamati ya usimamizi majukumu yako ni yapi ? na unatekeleza majukumu gani?***

1. Do you have the necessary skills to carry out the roles described above (question 2)? Please specify for which role you have the necessary skills and for which roles you do not have the necessary skills.

***Una utalaamu wa msingi juu ya nanmna ya kutekeleza majukumu uliyoainisha hapo juu? Taja ni utaalamu gani ulionao na usiokuwa nao katika utekelezaji wa majukumu yako.***

1. What do you think a health committee should be doing (in addition to the roles described

in 2)?

***Je , Unadhani ni kitu gani cha ziada ambacho Kamati ya usimamizi wa kituo ingefanya zaidi ya majukumu uliyoainisha hapo juu?***

1. The guidelines for health committees’ work says that health committees should carry out the following tasks (listed as A,B,C,D):

***Muongozo wa kamati ya usimamaizi ya kituo unaitaka kamati kutekeleza kazi zifuatazo (kama zilivyorodhehswa kwenywe vipengele A, B, C na D)***

1. “Provide governance as it relates to service provision within the facility/facilities”

***Kusimamia masuala ya utawala na uongozin katika kituo kulingana na miongozo ya utoaji wa huduma .***

1. How do you understand this task? Please describe in your own words:

***unalitekelezaje jukumu hili? Tafadhali elezea.***

1. Do you have the necessary skills to carry out this task? a)Yes b) No

***Je, umepata mafunzo yoyote ya kukuwezesha kutekeleza jukumu hili a)Ndio b) Hapana***

1. “Take steps to ensure that the needs, concerns and complaints of patients and the community are properly addressed by the management of the facility”

***Kuchukua hatua na kuhakikisha kuwa mahitaji, matakwa na malalamiko ya wagonjwa na jamii yanafanyiwa kazi ipasavyo na menejimenti ya kituo.***

1. How do you understand this task? Please describe in your own words:
2. ***je, unatekelezaje jukumu hili? Tafadhali eleza***

1. Do you have the necessary skills to carry out this task? a)Yes b)No c)N/A

***Je, umepatiwa mafunzo yoyote ya utekelezaji wa jukumu hili? a)Ndio b) Hapana***

1. “Foster community support for the initiatives and programmes of the facility”

***Kuhamasisha ushiriki wa jjamii katika miradi ya maendeleo ya kituo.***

1. How do you understand this task? Please describe in your own words:
2. ***Je, unatekelezaje jukumu hili? Tafadhali eleza***
3. Do you have the necessary skills to carry out this task? a)Yes b)No c)N/A

***Je, umepatiwa mafunzo yoyote ya utekelezaji wa jukumu hili?a)Ndio b) Hapana***

1. “Monitor the performance, effectiveness and efficiency of the facility/facilities”

***Kufuatilia utendaji, ufanisi na ubora wa kituo.***

1. How do you understand this task? Please describe in your own words.
2. ***je, unatekelezaje jukumu hili? Tafadhali elezea***
3. Do you have the necessary skills to carry out this task a)Yes b)No c)N/A

***Je, umepatiwa mafunzo yoyote ya utekelezaji wa jukumu hili?a)Ndio b) Hapana***

1. Please list the training that you have attended as part of a health committee in the following table:

***Tafadhali orodhesha idadi na aina ya mafunzo uliyowahi kupata katika nafasi yako ya mwenyeketi wa kamati ya usimamizi wa kituo***

| Training Course  ***Aina ya mafunzo*** | How long was the course?  ***Muda wa mafunzo*** | Who offered the course?  ***Aliyetoa mafunzo*** | Did you receive a certificate? (Yes or No)  ***Je ulipata cheti? (Ndio au Hapana)*** | How useful was the training?  (Refer to the key)  Mafunzo yalikuwa na manufaa kwa kiasi gani?  (Rejea ufunguo) | Please describe briefly why it was useful or not?  ***Eleza kwa kifupi namna gani yalikuwa na umuhimu au la*** |
| --- | --- | --- | --- | --- | --- |
|  |  |  |  |  |  |
|  |  |  |  |  |  |
|  |  |  |  |  |  |
|  |  |  |  |  |  |
|  |  |  |  |  |  |

Key/ Ufunguo:

1. Not useful/ Hayakuwa na manufaa
2. Slightly useful/ Yalikuwa na manufaa kiasi
3. Very useful/ Yalikuwa na manufaa sana
4. Which training course/s was/were the most useful you have received whilst a Health Committee member that you feel you are currently using in your role as a committee member?

***Ni mafunzo gani ambayo ulipatiwa katika kipindi chako cha kuwa mwenyekiti wa kamati ambayo unayatumia zaidi katika kutekeleza majukumu yako kwa sasa?***

1. What previous experiences have provided you with skills useful to be a health committee member and chairperson?

***Ni uzoefu gani ulio nao wa awali ambao umekupatia ujuzi wa kuwa mjumbe na mwenyekiti wa kamati ya usimamizi wa kituo hiki?***

1. When you joined the health committee, did you receive any orientation or induction? Please explain.

***Je, ulipochaguliwa kuwa mjumbe wa kamati ya usimamizi wa kituo, ulipatiwa mafunzo yoyote au maelezo ya utangulizi kabla ya kuanza utekelezaji wa majukumu yako? Tafadhali eleza.***

1. What training do you think an orientation and induction programme for new health committee members should include?

***Unadhani mafunzo gani au maelezo gani ya utangulizi ambayo mjumbe wa kamati ya usimamizi wa kituo anatakiwa apatiwe ?***

1. Have you been offered any training that you were unable to attend? A) Yes b) No

***Ulishawahi kupatiwa fursa ya mafunzo na ukashindwa kuhudhuria? A) Ndio b)Hapana.***

1. If you answered yes to question 12: what was the reason for not being able to attend the

training?

***Kama umejibu ndio kwenye swali la 11; ni sababu zipi zilikufanya ushindwe kuhudhuria mafunzo hayo?***

1. What are the roles for financial resources management for you as a chair of HFGC?

***Ni yapi majumu yako ya usimamizi wa fedha kama mwenyekiti wa kamati ya usimamizi wa kituo?***

1. Is there any challenges you are facing in the course of financial management practices?

***Je kuna changamoto gani ambazo unakumbana nazo kwenye usimamizi wa fedha?***

**Mechanisms of impact**

1. What is your relationship with health facility staff in management of health facility fund?

***Elezea uzoefu wa mahusiano kati yako na watumishi wa kituo katika usimamizi wa fedha za kituo?***

1. What is your relationship with CHMT members in the management health facility fund ?

***Elezea uzoefu wa mahusiano kati yako na timu ya usimamizi wa afya ya wilaya kwenye usimamizi wa fedha za kituo?***

1. What is your relationship with community in relation to facility management?

***Elezea uzoefu wa mahusiano yako na jamii katika usimamizi wa kituo?***

1. Has your role as chair of HFGC affected any of your routine activities? If so, how? How do you handle this?

***Katika majukumu yako kama mwenyekiti wa kituo yameathiri vipi kazi zako za kila siku?***

1. What is your convenience in the availability and management of resources at the facility level?

***Upi uzoefu wako katika upatikanaji na usimamizi wa rasilimali katika katika ngazi ya kituo.***

- 1. Drug supply management

***Usimamizi wa usambazaji wa dawa***

- 1. Equipment

***Vifaa tiba (mf. Vitanda, mizani nk)***

- 1. Staffing

***Watumishi***

1. What are the different projects are being implemented in this health facility?

***Ni miradi ipi ya maendeleo inayotekelzwa katika kituo hiki cha afya? (Mf. Mradi wa mama na mtoto, Malaria, UKIMWI na Kifua Kikuu)***

1. Are there any social economical factors that contribute or affect project implementation here?

Probes: cultural issues, political and policies.

***Je, kuna sababu zozote za kijamii, kisiasa na kiuchumi zinazochangia au kuathiri utekelezaji wa miradi hapa?***

***Chunguza; sababu za kitamaduni, kisiasana za kisera.***

1. In your experience, how can these challenges be addressed?

***Kwa uzoefu wako, ni njia gani zinaweza kutumika kutatua changamoto hizo?***

1. Do you have a Village Health Committee?

***Je mnakamati ya afya ya kijiji?***

1. If yes, how do you work with them?

***Kama ndio, mnashirikianaje katika kazi?***

1. Do you get remunerated with any incentives? If yes, What type?

***Je, huwa unapewa motisha yoyote katika kuyatekeleza majukumu yako? Kama ndio, ya aina gani?***

**Appendix No. 3 Questionnaire for patient exit interview**

***Kiambatanisho na.3 Dodoso la mgonjwa aliyemaliza huduma kituoni.***

Objective 3 (Impact assessment): To assess the effect of the DHFF program on responsiveness to the health system as perceived by the end users.

***Lengo Na.3 (Uhakiki wa matokeo): Kuhakiki matokeo ya mfumo wa kutoa fedha kwenye vituo moja moja kwenye mapokeo ya mfumo wa huduma za afya kwa wananchi.***

Serial No. [__|__|__|__]

***Namba ya kumbukumbu***

Name of the Region: _____________________________________

***Jina la Mkoa;***

Name of the District Council: ______________________________

***Jina la Wilaya:***

Ward _____________________________Village/Street _______________

***Kata ______________________________ Kijiji/ Mtaa________________***

Name of the Health Facility: _______________________________

***Jina la kituo:***

Type of Health Facility: 01= Dispensary [ ] 02= Health center [ ]

***Aina ya kituo: 01 = Zahanati [ ] 02 = Kituo cha afya [ ]***

Please put the appropriate number of a response in the given box

***Tafadhali weka namba ya jibu sahihi kwenye chumba ulichopewa***

| SN  ***Na.*** | Questions  ***Maswali*** | | Responses  ***Majibu*** | Code | Code |
| --- | --- | --- | --- | --- | --- |
| SECTION A: DEMOGRAPHIC INFORMATION  ***KIPENGELE A: TAARIFA ZA AWALI ZA MDODOSWAJI.*** | | | | | |
|  | (a)Sex  ***Jinsia*** | 1.Male  ***Mwanaume***  2.Female  ***Mwanamke*** | | 1  0 | [ ] |
|  | (b)How old are you?  ***Una umri gani?*** | 1. 15 – 24 years 2. 25 – 35 year 3. 36 – 44 year 4. 45 and above | | 1  2  3  4 |  |
|  | Marital status  ***Hali ya ndoa*** | 1.Married  ***Nina ndoa***  2.Cohabiting  ***Tunaishi pamoja***  3.Single  ***Sina ndoa***  4.Divorced/Separated  ***Mtalaka***  5.Widow/widowed  ***Mjane / Mgane*** | | 01  02  03  04  05 | [ ] |
|  | Highest level of education  ***Kiwango cha elimu*** | 1.Primary  ***Msingi***  2.Secondary  ***Sekondari***  3.Certificate  ***Astashahada***  4.Diploma  ***Stashahada***  5.Advanced diploma  ***Astashahada ya juu***  6.University degree  ***Shahada***  7.Masters  ***Shahada ya uzamili***  8. Others  ***Nyingine*** | | 1  2  3  4  5  6  7  8 | [ ] |
|  | What is the size of your family?  ***Familia yako inawatu wangapi?*** | 1.Below 3 members  ***Chini ya watu watatu***  2. 4 – 6 members  ***Watu 4 mpaka 6.***  3. Above 6  ***Zaidi ya watu 6*** | | 1  2  3 |  |
|  | What is the number of visits you have had to this facility before?  ***Umewahi kupata huduma mara ngapi katika kituo hiki?*** | 1.Twice  ***Mara mbili***  2.Thrice  ***Mara tatu***  3.Four times  ***Mara nne***  4.Five times  ***Mara tano***  5. More than five  ***Zaidi ya mara tano*** | | 01  02  03  04  05 | [ ] |
|  | How much distance would you have to undertake in order to reach health care?  ***Unatembea umbali gani kuifikia huduma ya afya?*** | 1. Within 5 kilometers   ***Ndani ya kilomita 5***   1. 5 – 10 kilometers   ***Kilomita 5 mpaka 10***   1. Above 10 kilometers   ***Juu ya kilomita 10*** | | 1  2  3 |  |

| Prompt to Attention (7)  ***Umakini*** | Very often (3)  ***Mara zote*** | Often (2)  ***Mara kwa mara*** | Slightly Often (1)  ***Mara chache*** | Never happens (0)  ***Haijawahi kutokea*** |
| --- | --- | --- | --- | --- |
| 1. How often did the health service providers listen to what you said with full attention during provision?   ***Nimara ngapi watoa huduma wa afya wamekusikiliza kwa umakini wakati wa kupata huduma?*** |  |  |  |  |
| 1. How often your statements were deeply understood by the health service providers?   ***Nimara ngapi maeezo yako yameeleweka vyema kwa watoa huduma wa afya wa kituo hiki?*** |  |  |  |  |
| 1. How often did health service providers spend enough time in asking you questions?   ***Mara ngapi mtoa huduma ametumia muda wa kutosha kukuuliza maswali juu ya ugojwa wako?*** |  |  |  |  |
| 1. How often the health service providers were accurately and actively in following up your treatment process?   ***Ni kwa kiwango gani watoa huduma wa afya wamekuwa makini katika kufuatilia matibabu yako ndani ya kituo cha kutoa huduma?*** |  |  |  |  |
| 5. The patients with similar needs are treated equally in the health units? |  |  |  |  |
| 6.Patients with un equal needs are treated equally in the health units? |  |  |  |  |
| 7.The health has always met my expectations |  |  |  |  |
| Respect to Dignity (3)  ***Utu*** | | | | |
| 1.How often did the health service providers show courtesy and affection towards you during service provision?  ***Ni mara ngapi watoa huduma wa afya wameonyesha heshima na upendo kwako wakati wa kupata huduma?*** |  |  |  |  |
| 2.How often did the health care workers paid attention specifically into your needs and characteristics?  ***Ni mara ngapi mtoa huduma amekuwa makini hasa kwa mahitaji yako na hali yako?*** |  |  |  |  |
| 3.How often is respect shown for the patient’s desire for privacy during treatment and examination? |  |  |  |  |
| Clear Communication (7)  ***Mawasiliano bayana*** | | | | |
| 1.How often did health care workers explain things in a way you could understand?  ***Ni mara ngapi watoa huduma wameeleza vyema mambo yanayokuhusu katika njia unayoielewa?*** |  |  |  |  |
| 2.How often health care workers explain things and issues related to your health in detail for you?  ***Ni kwa kiwango gani watoa huduma wa afya hueleza hali yako ya afya kiundani kwako?*** |  |  |  |  |
| 3.How would you rate your experience about how well you were treated as human during interection with the following service providers?  Nurses |  |  |  |  |
| 1. Laboratory staff |  |  |  |  |
| 1. Medical doctors/clinicians |  |  |  |  |
| 1. Security staff |  |  |  |  |
| 1. How wiould overall rate quality of interection at this health facility? |  |  |  |  |
| Autonomy (3)  ***Uhuru wa kufanya mahamuzi*** | No problem ***(3)***  ***Hakuna tatizo*** | Average  ***(2)***  ***Wastani*** | Big ***(1)***  ***Kubwa*** | Very big  ***(0)***  ***Kubwa sana*** |
| 1.How big a problem if any was it to get an appointment with the health care worker of your choice?  ***Ni kwa kiwango gani ulipata tatizo (kama lipo) la kupata fursa ya kukutana na mtuo huduma wa afya uliyemhitaji?*** |  |  |  |  |
| 2.How big a problem if any was it to use other health facility other than the one you usually went to?  ***Nikwa kiwango gani ulipata tatizo (kama lipo) kutumia kituo kingine cha huduma ya afya zaidi ya kile ambacho huenda kila wakati?*** |  |  |  |  |
| 3. I feel that my physicians have provided me choices and options |  |  |  |  |
| Access to Care (4)  ***Upatikanaji wa huduma*** | Waited for long time (above 30min)  (1)  ***Nilisubiri muda mrefu (zaidi ya dakika 30)*** | Average (up to 30min)  (2)  ***Wastani (mpaka dakika 30)*** | Waited for few minutes  (3)  ***Kusubiri kwa dakika chache*** | Serviced instantly  (4)  ***Nilipata huduma mara moja*** |
| 1.How long did you have to wait to get medical consultation from service provider?  ***Ulitumia muda gani kusubiri huduma ya afya?*** |  |  |  |  |
| 2.How long did you have to stay in the waiting room?  ***Ulisubiri muda gani katika chumba cha kusibiri kumuona mtoa huduma wa afya?*** |  |  |  |  |
| 3.How long did you have to stay at the pharmacy or dispensing area? |  |  |  |  |
| 4.How long did you have to stay waiting for laboratory services and results? |  |  |  |  |
| Confidentiality (3)  ***Usiri*** | | | | |
| 1.How often interviews remained confidential?  ***Kwa kiwango gani mahojiano yamekuwa ya usiri?*** |  |  |  |  |
| 2.Health care workers keep your personal information and records confidential?  ***Watoa huduma ya afya huweka taarifa zako binafsi kwa siri?*** |  |  |  |  |
| 3.Is the confidentiality maintained in this health facility? |  |  |  |  |
| **Basic Amenities**  ***Mahitaji muhimu*** | Strongly agree  (4)  ***Nakubali kabisa*** | Agree  (3)  ***Nakubali*** | Disagree  (2)  ***Sikubali*** | Strongly disagree  (1)  ***Sikubali kabisa*** |
| 1.I agree that this facility have enough buildings for service delivery  ***Nakubali kuwa kituo kina majengo ya kutosha ya kutoa huduma*** |  |  |  |  |
| 2.I agree that this facility have enough staffs to service patients  ***Nakubali kuwa hiki kituo kina watoa huduma wa kutosha kutoa huduma kwa wagonjwa*** |  |  |  |  |
| 3.I agree with the quality of direction aids of this facility  ***Nakubaliana na ubora wa vielelezo na miongozo iliyo kwenye kituo hiki.*** |  |  |  |  |
| 4.I agree with the cleanliness of this surroundings  ***Nakubaliana na hali ya usafi wa hiki kituo*** |  |  |  |  |
| 5.I agree with the waiting environment of this facility (waiting seats)  ***Nakubaliana na mazingira ya kusubiria huduma ya kituo hiki (ikiwa ni pamoja na viti)*** |  |  |  |  |
| 6.Are you convenient with the safety of service delivery environment in this facility?  ***Je unaridhishwa na mazingira ya usalama ya utoaji huduma wa hiki kituo?*** |  |  |  |  |
| 7. Access to clean water at health care units |  |  |  |  |
| 8. Cleaness of the toilets in the health care units |  |  |  |  |
| 9.Facilities for people with disabilities in the health care units |  |  |  |  |
| 10. The smell in the health care units |  |  |  |  |

# Appendix No. 4(a1) Questionnaire for Health Care Workers.

***Kiambatanisho Na.4(a) Dodoso la Watoa Huduma za Afya. .***

**Health service providers**

***Watoa huduma za Afya***

**Objective No.1;** To asses the Knowledge, Acceptability and Practice of Heath service providers towards DHFF program implémentation

Serial No. **[__|__|__|__]**

Name of the Region: _____________________________________

***Jina la Mkoa***

Name of the District Council: ______________________________

***Jina la Wilaya*** [_____________]

Name of the Health Facility: _______________________________

***Jina la kituo cha kutolea huduma***

Type of Health Facility: **01**= Dispensary/*Zahanati* **[ ] 02=** Health center/ *Kituo cha Afya* **[ ]**

***Aina ya Kituo*** [________]

**Please put the appropriate number of a response in the given *box.***

***Tafadhali jaza namba yenye jibu/majibu sahihi***

PART 1: HEALTH SERVICE PROVIDERS

***Sehemu ya kwanza:watoa Huduma***

| **SN** | **Questions/*Maswali*** | | **Responses /*Majibu*** | **Code** | **Code** |
| --- | --- | --- | --- | --- | --- |
| **SECTION A: DEMOGRAPHIC INFORMATION** | | | | | |
|  | (a)Sex/***Jinsi*** | 1.Male/***Mwanaume***  2.Female/ ***Mwanamke*** | | 01  02 | [ ] |
|  | How old are you?  ***Una umri gani?*** |  | |  |  |
|  | Marital status  ***Hali ya ndoa*** | 1.Married/***Nimeoa/Nimeolewa***  2.Cohabiting/***Tunaishi pamoja bila ndoa***  3.Single/ **Sijaoa/Sijaolewa**  4.Divorced/Separated/***Tumeachana***  5.Widow/widowed/***Mjane/Mgane*** | | 01  02  03  04  05 | [ ] |
|  | Highest level of education  ***Elimu yako*** | 1.Certificate/***cheti***  2.Diploma/***stashahada***  3.Advanced diploma/***stashahada ya juu***  4.University degree/ ***shahada***  5.Masters (MMED)/ ***shahada ya uzamili***  6. Masters (MPH)/***shahada y***a ***uzamivu ya utawala wa afya ya jamii***  7. Masters (MSc) *shahada ya uzamili ya sayansi*  8. Other (Mention)…..  ***Nyingine (Taja)……..*** | | 01  02  03  04  05  07  08 | [ ] |
|  | What is your cadre?  ***Taalum uliyosomea*** | 1.Enrolled Nurse  ***Nesi mwenye cheti***  2.Registered Nurse  ***Nesi mwenye astashahda***  3.Nurse Officer  ***Nesi mwenye shahada***  4.Assistant Clinical Officer  ***Tabibu msaidizi***  5.Clinical Officer  ***Tabibu***  6.Assistant Medical Officer  ***Msaidizi wa Daktari***  7.Medical Officer/Dental Officer  ***Daktari***  8.Medical specialist (MMED)  ***Daktari bingwa***  ***9.***Assistant Accountant  ***Mhasibu Msaidizi***  10. Health Facility Governance Committee Chair/ Member  ***Mwenyekiti wa Kamati ya Usimamizi wa Kituo.*** | | 01  02  03  04  05  06  07  08  09  10 | [ ] |
|  | How long have you been working in your current job/position? [Clinician]/ [nurse] *(record number of years or term served)*  ***Je! umefanya kazi kwa muda gani?*** |  | |  | [ ] |
|  | What is your position at this health facility?  ***Unacheo gani katika kituo chako cha kazi?*** | 1. Health service provider 2. Matron 3. In charge 4. Assistant Account 5. HFGC Chair/ Member. 6. Other (Mention)___________ | | 01  02  03  04  05  06 | [ ] |
|  | **Assessment of Acceptability of DHFF Program** | | | |  |
|  | In general, do you like the way DHFF program operates? *(If the answer is no skip the following questions 9-19, 27, 28, 42 – 44, 46, and 49)* | 1. No  2. Yes | | 01  02 | [ ] |
|  | How do you feel about the introduction of DHFF program? | 1.Uncomfortable  2. Comfortable | | 01  02 | [ ] |
|  | Do you agree that, DHFF is beneficial? | 1. No  2. Yes | | 01  02 | [ ] |
|  | If the answer is NO, pick the reasons from the listprovided. | 1. Work load 2. Skills on financial management 3. Unavailability of working guidelines and tools | |  |  |
|  | In the course of introduction of DHFF, were you worried about challenge associated with increase of work load? | 1. No  2. Yes | | 01  02 | [ ] |
|  | In the course of introduction of DHFF, were you worried about challenge associated with competency in running the program properly? | 1. No  2. Yes | | 01  02 | [ ] |
|  | In the course of introduction of DHFF, were you worried about challenges associated with availability of working tools? | 1. No  2. Yes | | 01  02 | [ ] |
|  | In the course of introduction of DHFF were you worried about challenges associated with reporting process? | 1. No  2. Yes | | 01  02 | [ ] |
|  | **Knowledge Assesssment** | | | |  |
|  | Have you ever heard about DHFF program?  ***Umeawhi kusikia kuhusu mpango wa kupeleka fedha moja kwa moja katika kituo cha kutolea huduma (DHFF)?*** | 1. No / ***Hapana*** 2. Yes / ***Ndio*** | | 01  02 | [ ] |
|  | Where did you heard about DHFF program?  ***Uliskia kuhusu mpango huu wapi?*** | 1. On the training  ***Kwenye mafunzo***  2. From In charge of HF  ***Kwa mfawidhi wa Kituo***  3. From other staffs.  ***Kutoka kwa watumishi wengine.***  4. Somewhere else (mention)……………..  ***Mahali pengine (Taja)……..*** | | 01  02  03  04 |  |
|  | What minimum requirements do you have for the implementation of DHFF at facilities? *(multiple selection)*  *Assess the performanc of the given variable, if the facility missed one of them then its lack minimum requirements.*  ***Je, Ni mahitaji gani ya msingi mliyo nayo kwa ajili ya utekelzaji wa mpango wa kupeleka fedha moja kwa moja katika vituo vya kutolea huduma (DHFF).*** | 1. Health facility accounts   ***Akaunti ya wa kituo***   1. At least one skilled personnel   ***Angalau mtaalam mmoja***   1. Active HFGC   ***Kuwepo kwa kamati ya usimamizi wa kituo inayofanya kazi.***   1. Others (mention)________   ***Nyingine (taja)….*** | |  |  |
|  | (FoI) Were you trained on FFARS and DHFF program  ***Umawahi kupata mafunzo ya mfumo wa Usimamizi wa fedha na kutolea taarifa wa za vituo (FFARS) pamoja n***a  ***mpango wa kupeleka fedha moja kwa moja katika kituo cha kutolea huduma (DHFF)?*** | 1. No **/ *Hpana*** 2. Yes / ***Ndio*** | | 01  02 | [ ] |
|  | **Assessment of Practice of DHFF Program** | | | |  |
|  | (FoI) Do you have active HFGC? (up to date list of members)  ***Mna kamati hai ya usimamizi ya kituo?*** | 1. No/***Hapana*** 2. Yes/**N*ndio*** 3. I don’t know **/ Sijui** | | 1  0  2 | [ ] |
|  | Do you have HFGC working guide?  ***Una muongozo wa wa namna ya kufanya kazi kwenye kamati ya usimamizi?*** | 1. No 2. Yes 3. I don’t know **/ Sijui** | | 1  0  2 |  |
|  | Do you convene quarterly HFGC meetings?  ***Je? Mnafanya vikao vya robo vya kamati ya usimamizi wa kituo?*** | 1. No /Hapana 2. Yes / Ndio 3. I don’t know **/ Sijui** | | 1  0  2 | [ ] |
|  | Do you keep minutes of HFGC meetings.  ***Mnatunza miutahsari ya vikao vya kamati ya usimamizi wa kituo?*** | 1. No/ ***Hapana*** 2. Yes (Please show)/***Ndio (angalia kuhakiki)*** 3. I don’t know **/ Sijui** | | 1  0  2 |  |
|  | Does your facility have a MoFP approved account?  ***Kituo chenu kina akaunti iliyothibitishwa na Wizara ya Fedha?*** | 1. No /***Hapana*** 2. Yes / ***Ndio*** 3. I don’t know **/ Sijui** | | 01  0  2 | [ ] |
|  | (FoI) Do you have an assistant accountant in this HF?/ Do you receive satellite accounting service?  ***Mna muhasibu msaidizi wa kituo?/ Mpata Huduma ya msaada wa masuala ya fedha toka kwa mhasibu wa kituo kingine?*** | 1. No /***Ndio*** 2. Yes /***Hapana*** 3. I don’t know **/ Sijui** | | 1  0  2 | [ ] |
| 27. | (FoI) Do you have Guidelines and operational manuals for DHFF and FFARS  **Mna miongozo ya kuendeshea *mfumo wa Usimamizi wa fedha na kutolea taarifa wa za vituo (FFARS) pamoja n***a  ***mpango wa kupeleka fedha moja kwa moja katika kituo cha kutolea huduma (DHFF)?*** | 1. No / ***Hapana*** 2. Yes / ***Ndio*** 3. I don’t know **/ Sijui** | | 1  0  2 | [ ] |
| 28. | (FoI) Do you have PFM tools to manage DHFF *(observe the availability of basic accounting and records to be maintained at heath facilities attached at the end of the checklist)*  ***Mna zana zana usimamizi wa fedha za serikali( PFM tools) kwa ajili ya mpango wa kupeleka fedha moja kwa moja katika kituo cha kutolea huduma (DHFF)? `Angalia kama zipo`*** | 1. No / ***Hapana*** 2. Yes / ***Ndio*** 3. I don’t know **/ Sijui** | | 1  0  2 | [ ] |
| 29. HCWs only. | How many times do you convene HF Management meetings and then give various response options per quarter?  Ni ***mara ngapi mnakaa vikao vya Menejimenti ya kituo kwa robo mwaka***? |  | |  | [ ] |
| 30. HCWs only. | What are the dates of the last two HF Management meetings you have conducted?  ***Taja, tarehe za vikao viwili vya mwisho vya Menejimenti ya Kituo.*** |  | |  |  |
| 31. | Do you have a HF Quality Improvement Plan (QIP)?  ***Mna mpango wa uboshaji wa huduma?*** | 1. No /***Hapana*** 2. Yes / ***Ndio*** 3. I don’t know **/ Sijui** | | 1  0  2 | [ ] |
| 32.HFGC only. | Do you endorse a Quality Improvement Plan (QIP) for your health facility?  ***Je, huwa unaidhinisha mpango wa uboreshaji wa huduma wa kituo?*** | - - 1. No /Hapana     2. I don’t know   sijui  3. Yes / Ndio | | 1  0  0.5 | [ ] |
| 33. | Do you have Annual Health Facility Plan Guideline?  ***Je, Mnamuongozo wa kuandaa mpango kazi wa kituo?*** | 1. No/***Hapana***  2. I don’t Know/ ***Sijui***  3. Yes/ ***Ndio*** | |  |  |
| 34. | Do you have annual HFP?  ***Mna mpango wa kituo wa mwaka***? | 1. No /***Hapana*** 2. Yes / ***Ndio*** | | 01  02 | [ ] |
| 35. | Budget ceilings are received on time (before November)  ***Taarifa za ukomo wa bajeti hupatikana kwa wakati*** | 1. No /***Hapana*** 2. Yes / ***Ndio*** 3. I don’t know **/ Sijui** | | 1  0  2 | [ ] |
| 36. | Head teacher from a school near by facility take part in the planning team.  ***Mwalimu Mkuu au Mwalimu mwingine hushiriki katika zoezi la uandaaji wa mpango wa Kituo.*** | 1. No /***Hapana*** 2. Yes / ***Ndio*** 3. I don’t know **/ Sijui** | | 1  0  2 | [ ] |
| 37. | Always Planning is coordinated by representative from CHMT and Technical.  ***Mchakato wa uandaaji wa mpango wa kiuo huratibiwa na Mjumbe kutoka CHM na Kamati ya Ufundi au Mratibu wa Kanda.*** | 1. No /***Hapana*** 2. Yes / ***Ndio*** | | 01  02 | [ ] |
| 38. | Member from Village/Ward Development Committee if not a member of HFGC take part in the planning team of the health facility.  ***Mjumbe kutoka kamati ya kijiji/ kata kama sio miongoni mwa wajumbe wa kamati ya usimamizi wa kituo hushiriki katika mchakato wa kuandaa mpango wa kituo.*** | 1. No /***Hapana*** 2. Yes / ***Ndio*** | | 01  02 | [ ] |
| 39. | Have you received funds for DHFF program*(if the answer is no skip questions 40, 42,43,45,46, ,47,48 and 49,)*  ***Mmeshapata fedha za mpango wa kupeleka fedha moja kwa moja katika kituo cha kutolea huduma (DHFF)?***  ***(Kama hapana neda swali la 40,42,43,45,46,47,48 na 49*)** | 1. No /***Hapana*** 2. Yes / ***Ndio*** 3. I don’t know **/ Sijui** | | 1  0  2 | [ ] |
| 40 | When did you receive money for this quarter?  ***Mlipokea lini fedha ya robo hii ya mwaka?*** | 1. Before 14^th^ of first month of the following quarter.  ***Kabla ya tarehe 14 ya mwezi wa kwanza wa robo ya mwaka iliyofuata.***  2. On 14^th^ of first month of the following quarter.  ***Manamo tarehe 14 ya mwezi wa kwanza wa robo ya mwaka iliyofuata.***  3. After 14^th^ of first month of the following quarter.  ***Baada ya tarehe 14 ya mwezi wa kwanza wa robo ya mwaka iliyofuata.***  4. Not yet received***/ Bado hatujapata.*** | | 01  02  03  04 | [ ] |
| 41 | If No Why?  ***Kama hapana kwa nini?*** | 1. We are yet to receive funding   ***Bado hatujaingiziwa fedha***   1. We are yet to be trained   ***Bado hatujapata mafunzo***   1. We are not aware at all   ***Hatuelewi*** | | 01  02  03 |  |
| 42. | What are the challenges you are facing in the course of implementing DHFF? (Request for Qualitative semi structured interview guide)  ***Je! kuna changamoto gani mnapata wakati wa utekelezaji wa mpango wa kupeleka fedha moja kwa moja katika kituo cha kutolea huduma (DHFF)*** | 1. Inadequate financial management skills.   ***Uwezo mdogo wa usimamizi wa fedha***   1. Inadequate transparent among team.   ***Kukosekana kwa uwazi***   1. Inadequate supportive supervision.   ***Usimamizi shirikishi usi wa kuridhisha***   1. Inadequate health service providers.   ***Upungufu wa watoa huduma***   1. Unsupportive relationship with HFGC. 2. Poor coordination from district level. 3. Inadequate availability of working tools. 4. Inadequate availability of accounting services. 5. Program complexity. 6. Political interfearance. 7. No challenges. 8. Other (Mention)   ***Mengineyo (taja)*** | |  | [ ] |
| 43. | In case of any challenges in the course of implementing the program, where do you report first? | 1. DMO 2. District DHFF coordinator 3. Others……(mention) | |  |  |
| 44. | What has been helpful to you in achieving DHFF goals in this Health Facility?  ***Unadhani kitu gani kimewezesha nyinyi kufanikiwa kutekeleza mpango huu wa DHFF katika kituo hiki?*** | 1. Supportive supervision   ***Usimamizi shirikishai***   1. Provision of working tools   ***Kuongeza vitendea kazi***   1. Employment of other staff   ***Kuajiri watumishi***   1. Others (mention)________   ***(Mengine taja)*** | |  |  |
| 45. | When did you receive your last supportive supervision from CHMT?  ***Ni lini ulifanyika usimamizi shirikishi wa timu ya uisimamizi wa Afya ya Wilaya (CHMT)?*** | 1. Last Quarter   ***Robo iliyopita***   1. This quarter   ***Robo hii***   1. Not yet (skip question 46)   ***Bado haujafanyika*** | | 01  02  03 | [ ] |
| 46. | During the supportive supervision did you discuss issues of DHFF implementation?  ***Katika kipindi cha ukaguzi mlijadili masuala ya DHFF?*** | 1. No 2. Yes | |  |  |
| 47. | Have you received any feedback on the previous supportive supervision?  ***Umewahi kupata mrejesho wa*** ***usimamizi shirikishi wa timu ya uisimamizi wa Afya ya Wilaya (CHMT)?*** | 1. No /***Hapana*** 2. Yes ***/ Ndio*** | | 01  02 | [ ] |
| 48. | Who is making decision of your routine Health Facility activities?  ***Nani anafanya maamuzi ya mpango wa kituo chenu?*** | 1. Matron 2. In charge 3. Any one 4. All of us through meetings. 5. Other (mention)________ | | 01  02  03  04  05 | [ ] |
| 49. | (FoI) Do you play an active role (*in charge*) / get involved *(health care workers)/* endorse *(HFGC)* (in) DHFF program implementation decision making?  ***Je unashiriki kikamilifu (mfawidhi wa kituo) / unashirikishwa kikamilifu (wahudumu wa kituo) katika maamuzi ya utekelezaji wa mpango wa kupeleka fedha moja kwa moja katika kituo cha kutolea huduma (DHFF)*** | 1. No /***Hapana*** 2. Yes / ***Ndio*** | | 01  02 | [ ] |
| 50. | What are the sources of your Health facility fund?  *(Multiple selection)*  ***Vyanzo vya fedha za kituo ni zipi?*** | 1. Health Basket Fund 2. Results Based Financing 3. Cost Sharing/ ***uchangiaji*** 4. Receipt in Kind 5. Council own sources   ***Mapato ya ndani***   1. Local Government capital Development Grant 2. Community Health Fund/TIKA 3. Implementing partner’s money 4. Other (mention)----- | | 01  02  03  04  05  06  07  08  09 | [ ] |
| 51 | Did you receive the whole amount of funds as requested in your last Financial Year Budget?  ***Kwa mwaka wa fedha uliopita mlipokea kiasi chote cha fedha mlichoomba kulingana na bajeti yenu ya kituo?*** | 1. No/***Hapana*** 2. I don’t know/ ***Sijui*** 3. Yes/ ***Ndio*** | |  |  |
| 52. | How frequently do you submit your Financial and Technical report on DHFF program implementation to the district level annualy?  ***Je, huwa mnapeleke taarifa ya utekelezaji wa mpango wa DFF wilayani kila baada ya muda gani?*** | 1. Every month   ***Kila mwezi***   1. After two months   ***Kila baada ya miezi miwili***   1. Every Three months   ***Kila baada ya miezi mitatu***   1. Other   ***Wakati mwingine taja*** | | 01.  02.  03.  04. | [ ] |
| 53. | When was your last report submission the (in question 50)? (Probe date and check where it falls along the answers provided)  ***Ni lini Mara ya mwisho kutuma taarifa zilizotajwa hapo juu? (Chunguza tarehe na onyesha inapopatikana katika majibu yaliyotolewa).*** | 1. Within seven days after the end of the quarter.  2. After seven days following end of the quarter.  3. I don’t know **/ Sijui** | | 01  02  03 | [ ] |
| 54. | Do you know Village Health Committee? *(If the answer is no skip questions 56 – 57)*  ***Unaifahamu kamati ya afya ya kijiji?*** | 1. No / ***Hapana*** 2. Yes / ***Ndio*** | | 01  02 | [ ] |
| 55. | If Yes, are you a member of that committee?  ***Kama ndio wewe ni mjumbe wa kamati hiyo?*** | 1. No / ***Hapana*** 2. Yes / ***Ndio*** | | 01  02 | [ ] |
| 56. | Have you ever attended in their meetings?  ***Umewahimkuhudhuria vikao hivyo?*** | 1. No / ***Hapana*** 2. Yes / ***Ndio*** | | 01  02 | [ ] |
| 57. | Do you work with Village Health Committee?  ***Unafanya kazi na kamati ya afya ya kijiji?*** | 1. No / ***Hapana*** 2. Yes / ***Ndio*** | | 01  02 | [ ] |
| 58. | What other programs apart form DHFF are implemented in this facility? (*List them*)  ***Ni programu gani nyingine za uboresha huduma za afya zinatekelezwa kwenye kituo hiki? (Taja)*** |  | |  |  |
|  | **Reccomendations for Improving Program Implementation** | | | |  |
| 59 | Which approach/ways/ means will you prefer to be used in submitting your reports?  ***Ni njia gani ungependelea kutumia wakati wa kutuma taarifa?*** | 1. Monthly submission as DHIS2   ***Kila mwezi kama kwenye***  ***mfumo wa DHIS2***   1. Weekly through text messages via mobile phones   ***Kwa ujumbe wa simu kila***  ***wiki***   1. On quarterly basis like any other reports for technical and financial   ***Kwa kila robo***   1. Other (mention)   ***Nyingine (taja)….*** | | 01  02  03  04 | [ ] |

**Appendix 4 (a2)**

**Direct Health Facility Financing (DHFF): Process Monitoring, Health Facility In charge, Indepth Interview Guide.**

**Objectives**

- Assess to what extent the HF team is fulfilling its role, and whether this has changed with DHFF program.
- Document contextual factors that might affect intervention impact.

**Questions**

1. How long have you been working in this facility?
2. How do you know about DHFF program?
3. What is the main purpose of the DHFF program?
4. What issues to be addressed to facilitate achievemens of the DHFF program?
5. Which steps did you undertake after the introduction of DHFF program in this facility? If Yes, Probe measures undertaken (*They must include; Meeting with HFGC, HF providers meeting, Meeting with Ward/Village council)*
6. What are your roles as HF incharge in implementation of DHFF program?
7. What are the roles of the HF team as far as the DHFF program is concerned?
8. Does the facility have an annual work plan and DHFF quarterly plans? If so, how was this developed? How were you involved? What factors were considered to develop the plan? *[****Note****: use of data from HMIS for planning]*
9. What activities have you conducted as a HF team, as part of the DHFF program? (Probe: Have you received any incentives/allowances to meet?)
10. What measures do you take to ensure effective and efficient implementation of the DHFF program? (Probe issues related to participatory decision making, consultation, mentorship etc).
11. Are there any benefits from the implementation of DHFF? If Yes, Mention them.
12. Are there any other factors beyond the facility that contribute or affect DHFF implementation here? Probes: cultural issues, policies and Political issues.
13. What other programs apart from DHFF are being implemented in this health facility? How do those programs relate to DHFF?
14. What is your experience with the implementation of DHFF program? (Probe issues related to easiness of compliance).
15. In general, how do you like the way DHFF program operates? What do you like and dislike?
16. Can you tell me something about the Village Health Committee? Probe more on the composition, function and their mandate.
17. How do you work with them?

**Thanks for your time!**

**Documents to be requested:**

- HFGC meeting minutes

**Appendix 4 (b2)**

**Direct Health Facility Financing (DHFF) program implementation in Tanzania: Process evaluation Interview Guide**

**HFGCs**

**Objectives**

- Assess DHFF implementation progress
- Generate and investigate hypotheses on individual-level changes and changes in relationships between actors/levels (District and Facility Level)from DHFF
- Document contextual factors that might affect intervention impact

**Icebreaker**

1. How long have you been working as a chair of HFGC?
2. What are your roles as a Chair to the HFGCs? What roles do you provide?

**Theory of change**

1. What do you know about DHFF program? What is the main purpose of the DHFF program?
2. What are your roles as a chair in DHFF program implementation?
3. What issues to be addressed to facilitate achievemens of the DHFF program? (Probe more about the activities)
4. How do you communicate back to community about the implementation of DHFF in this facility?

**Context**

1. What other programs apart from DHFF program are being implemented in this facility?
2. In your experience, what have been the challenges with DHFF program implementation? How could it work better?
3. Are there any other factors beyond the facility that contribute or affect DHFF implementation here? Probes: cultural issues, policies and Political issues.
4. Do you have a Village Health Committee? If yes, how do you work with them?
5. Have HFGC team, linked to this facility received any bonus payment? If so, how many cycles? For how much?

**Thanks for your time!**

**Appendix 4 (C2)**

**In-depth interview guide for DMO and DHFF Coordinator.**

This interview should be conducted with the District DHFF Coordinator. For each interview, say this information at the start of digital recording and write this information at the top of the transcript.

First I will ask a few general questions about you, your background and your employment

| Question | Response |
| --- | --- |
| DO NOT ASK .Indicate the sex of the respondent? | Female or Male |
| What is our current job title? | ................................................................ |
| What is the highest level of education you have completed? | ................................................................. |
| Do you have any medical qualification? If yes what is it? | .......................................................... |
| How many years have you been working? | ( ) ( ) years |
| How many years have you been working in this position? | ( ) ( ) years |

**Theme 1. How DHFF will affect health care system**

I would like to ask you about how DHFF program might change health system in this district.

1. Does introduction of DHFF program has effect on your execution of your daily activities at the health facility and your role? If yes how and If no How?

Probes: new policies, awareness, effect on Planning and Budgeting, Resources availability. Are there any challenges you are facing in the course of implementing DHFF program?

1. Do you discuss issues of DHFF at the CHMT Meetings?
2. What are the role of CHSB in the implementation of DHFF?

**Theme 2. Acceptability of DHFF program**

1. What are your views about the DHFF program?
2. Please describe to me the process from start to finish of how DHFF program works (How DHFF work from the facility to the national level)
3. What are the benefits of the DHFF program?
4. How do you support HF in planning process?
5. How do health service providers respond to the available DHFF guideline?
6. How do you track the performance of the DHFF program in your Health Facilities?
7. How RHMT support you in the implementation of DHFF program? (Probe for frequency?)
8. Which steps did you undertake during introduction of DHFF program in district? Probe measures undertaken (*They must include; Meeting with HFGC, HF providers meeting, Meeting with Ward/Village council)*
9. What are the benefits associated with implementation of DHFF in your district?
10. Which approach will you prefer the HF to submit DHFF implementation reports?
11. Do budget ceilings reveled before November of every year/ If Yes/No, do you feel of any other month to be suitable for ceilings submission and why?
12. How did you handle the transaction of disbursement of funds from the previous practice to the DHFF program implementations?
13. What are your accountability options to ensure that DHFF program is implemented as per design/expectations?
14. What do you think should be done to make DHFF program successful (probe reasons)
15. Imagine that you are the Minister of Heath Community Development, Gender, Elderly and Children instilled to decide whether or not to continue with the implementation of DHFF program. What would you do?

- If says to continue, ask if they would change anything
- If says to stop, ask why

1. Can you tell me something about the Village Health Committee? Probe more on the composition, function and their mandate.
2. How do you support them?
3. Is there anyone else who you think it would it be important for me to speak to about the program?

**Thanks for your time!**

**Appendix 4 (c3)**

**Direct Health Facility Financing (DHFF): Process Monitoring, Health Facility (HF) team Focus Group Discussion (FGD) Guide**

**Objectives**

- Assess to what extent the HF team is fulfilling its role, and whether this has changed with DHFF program
- Document contextual factors that might affect intervention impact

**Questions**

1. How long have you been workers of this facility?
2. What are your roles of your HF team as far as the DHFF program is concerned?
3. In your opinion, what is the main purpose of the DHFF program?
4. How do you think the program will bring about that change or impact? Through which activities?
5. What activities have you conducted as a HF team, as part of the DHFF program? Probe: Have you received any incentives/allowances to meet?
6. Does the facility have an annual plan and DHFF quarterly plans? If so, how was this developed? How were you involved? What factors were considered? *[****Note****: use of data from HMIS for planning]*
7. How have you been involved on the use of these funds? Were you satisfied with this involvement? Why/why not? Who else was involved? (Probe: for HWs)
8. Has the implementation of DHFF affected the way staff work at this facility?

Probes: Relationships between staff; Motivation, absenteeism

1. Any changes in how the health facility operates? What do you think has contributed to these changes? (Probe: resources, incentives, financial autonomy, supervision, attention to concrete goals)
2. Any change in what health workers does or do not do? What do you think has contributed to these changes? (Probe: resources, incentives, financial autonomy, supervision, and attention to concrete goals).
3. Have there been any changes in your relationship with the CHMT or the Community? [***Note:*** *Interest in impact on referrals*]
4. Are there any challenges you are facing in the course of implementing DHFF program?
5. Have you observed any changes in the availability and management of resources at the facility level?
   1. Drug supply management
   2. Equipment
   3. Staffing
6. Are you aware of how your facility is performing in terms of maternal and Child health indicators relative to others in the district? If so, how did you become aware of this and how do you feel about this? *[****Note:*** *to check if receiving feedback from verification]*
7. What would you say about access and service utilization by the community since the start of DHFF program? (Probe: what changes have you observed? What led to these changes?)
8. What other programs apart from DHFF are being implemented in this health facility?
9. Are there any other factors beyond the facility that contribute or affect DHFF implementation here? Probes: cultural issues, policies.
10. In your experience, what have been the biggest challenges with DHFF implementation?
11. Are you aware of Village Health Committee?
12. How do you work with them?

**Thanks for your time**

**Documents to be requested:**

- HFGC meeting minutes
